# In silico design of bivalent multi-epitope mRNA vaccine candidates against *Mycoplasma pneumoniae* and *Chlamydia pneumoniae*

**DOI:** 10.1186/s12866-026-05473-y

**Published:** 2026-08-06

**Authors:** Xu Wu, Yizhong Xu, Weiwei Zhao, Rongliu Qin, Yuying Luo, Yanqun Hou, Ziyou Zhou, Shiyang Ma, Fei Zhu, Pinhua Pan

**Affiliations:** 1 Department of Respiratory Medicine, Center of Respiratory Medicine, Clinical Research Center for Respiratory Disease, Xiangya Hospital, National Key Clinical Specialty, Branch of National, Central South University, Changsha, 410008 Hunan China; 2 Department of Respiratory and Critical Care Medicine, General Hospital of Beitun City, Xinjiang Production and Construction Corps, 10th Division, No. 979 Bowang East Street, Beitun, 836099 Xinjiang China; 3 Clinical Research Center for Respiratory Diseases in Hunan Province, Hunan Engineering Research Center for Intelligent Diagnosis and Treatment of Respiratory Disease, Xiangya Hospital, Central South University, Changsha, 410008 Hunan China; 4 National Clinical Research Center for Geriatric Disease, Xiangya Hospital, Central South University, Changsha, 410008 Hunan China; 5 Furong Laboratory, Changsha, 410078 Hunan China

**Keywords:** *Mycoplasma pneumoniae*, *Chlamydia pneumoniae*, Immunoinformatics, Reverse vaccinology, Bivalent multi-epitope mRNA vaccine, Population coverage, Molecular dynamics simulation, TLR2

## Abstract

**Background:**

Community-acquired pneumonia (CAP) remains a major global health concern. *Mycoplasma pneumoniae* (*M. pneumoniae*) and *Chlamydia pneumoniae* (*C. pneumoniae*) are important atypical CAP pathogens, and reported coinfection may complicate diagnosis and management. However, no licensed vaccine simultaneously targeting both pathogens is currently available. In this context, mRNA-based multi-epitope vaccines represent a promising platform for bivalent vaccine design because they allow the integration of multiple epitopes into a single construct and may support both humoral and cellular immune responses. This study aimed to design bivalent multi-epitope mRNA vaccine candidates against *M. pneumoniae* and *C. pneumoniae* using an immunoinformatics-based strategy.

**Methods:**

Conserved cytotoxic T-lymphocyte (CTL), helper T-lymphocyte (HTL), and linear B lymphocyte (LBL) epitopes were screened from selected antigenic proteins. Two constructs, MCV1 and MCV2, were assembled with distinct N-terminal immunostimulatory or helper-epitope modules and evaluated for antigenicity, safety, physicochemical and structural properties, receptor docking, three independent 100-ns molecular dynamics simulations, replicate-level MM-PBSA, immune simulation, population coverage, human codon optimization, and RNA secondary structure.

**Results:**

A total of 11 CTL, 5 HTL, and 14 LBL epitopes with ≥ 90% conservancy among the analyzed strain sequences were selected. Both core constructs were predicted to be antigenic, non-toxic, non-allergenic, and structurally acceptable. HADDOCK refinement yielded cluster-level scores of − 394.3 ± 7.3 for MCV1–TLR2 and − 399.0 ± 5.5 for the exploratory MCV2–TLR4 complex. Across three independent trajectories, MM-PBSA total binding free energies were − 125.19 ± 20.13 and − 126.87 ± 12.73 kcal/mol, respectively. C-ImmSim generated modeled profiles involving humoral and cellular immune components, and the estimated global population coverage was 97.90%. Human codon optimization yielded codon adaptation index values of 0.92 and 0.91 and GC contents of 61.06% and 60.94% for MCV1 and MCV2, respectively.

**Conclusion:**

In this in silico study, two bivalent multi-epitope mRNA vaccine candidates against *M. pneumoniae* and *C. pneumoniae* were computationally prioritized. These findings provide a theoretical basis for subsequent experimental validation, while in vitro and in vivo studies are still required to verify their actual immunogenicity, protective efficacy, and safety.

**Supplementary Information:**

The online version contains supplementary material available at https://doi.org/10.1186/s12866-026-05473-y.

## Background

*M. pneumoniae* and *C. pneumoniae* are important atypical bacterial pathogens that contribute substantially to CAP and other respiratory tract infections worldwide [1]. *M. pneumoniae* is a major cause of CAP, particularly in school-age children and adolescents, whereas *C. pneumoniae* is associated with both acute and persistent respiratory infections across different age groups [2, 3]. Together, these pathogens impose a considerable clinical and public health burden, especially in resource-limited settings, while effective preventive strategies are still lacking.

The prevention and clinical management of infections caused by these two pathogens remain challenging. Infections caused by *M. pneumoniae* and *C. pneumoniae* may complicate early etiological diagnosis because of overlapping clinical manifestations and heterogeneous disease courses, potentially delaying timely targeted treatment [4]. In addition, antibiotic-based management has important limitations. *M. pneumoniae* is intrinsically insensitive to cell wall-targeting antibiotics, such as β-lactams, because it lacks a typical bacterial cell wall, and the increasing prevalence of macrolide-resistant *M. pneumoniae* has further reduced treatment effectiveness in several regions worldwide [5, 6]. *C. pneumoniae* may establish persistent infection in the respiratory tract and has been associated with recurrent or prolonged respiratory symptoms [3]. Collectively, these factors indicate that reliance on antimicrobial therapy alone is insufficient for long-term prevention and control of these two pathogens, supporting the need for vaccine-based preventive strategies.

However, no licensed vaccines with proven clinical efficacy against *M. pneumoniae* or *C. pneumoniae* are currently available globally [7, 8]. Traditional vaccine development for these pathogens has faced challenges related to safety, breadth of protection, and translational efficacy [7, 8]. Notably, most published immunoinformatics-based vaccine studies for these two pathogens have focused on single-pathogen, monovalent designs, and, to our knowledge, no bivalent multi-epitope mRNA vaccine simultaneously targeting *M. pneumoniae* and *C. pneumoniae* has been reported to date [9, 10]. In this context, mRNA-based multi-epitope vaccines represent a promising platform because they enable the rational integration of multiple conserved epitopes from different pathogens into a single construct, offer flexibility for combined antigen design, and may facilitate the induction of both humoral and cellular immune responses [11]. These features make this platform suitable for the development of a bivalent vaccine targeting these two clinically relevant CAP pathogens.

Clinically, the rationale for a combined construct is not that coinfection is uniformly frequent, but that both organisms contribute to atypical CAP, can produce overlapping clinical syndromes, and may be difficult to distinguish during the early disease course. A single modular platform incorporating conserved antigens from both pathogens may therefore provide a practical framework for combined preventive development while reducing duplication in construct design and downstream validation.

Based on this rationale, we aimed to design and computationally evaluate a bivalent multi-epitope mRNA vaccine candidate against *M. pneumoniae* and *C. pneumoniae*. Systematic immunoinformatics approaches were applied to identify CTL, HTL, and LBL epitopes from five selected antigenic proteins. All selected targets have well-documented biological and immunological relevance and meet the critical criteria for vaccine targets in our pre-screening bioinformatic analyses, including favorable subcellular localization, non-toxicity, and low allergenicity.

These targets include the P1 adhesin (P1) and P30 adhesin (P30) from *M. pneumoniae*, two surface-localized adhesion molecules that are essential for mediating pathogen attachment to human respiratory epithelial cells, subsequent respiratory tract colonization, and the induction of host adaptive immune responses [12]; and the major outer membrane protein (MOMP), outer membrane complex protein A (OmcA), and outer membrane complex protein B (OmcB) from *C. pneumoniae*. MOMP is a major outer membrane antigen, whereas OmcA and OmcB are outer membrane complex-associated proteins that contribute to outer membrane organization and pathogen–host interaction [13].

Subsequently, multi-epitope vaccine constructs were assembled and evaluated in silico for antigenicity, safety profiles, physicochemical and structural characteristics, predicted receptor-interaction profiles, and simulated immune-response outputs. This study provides a computational foundation for the further experimental development of a combined vaccine against these two clinically important respiratory pathogens.

## Methods

### Screening and validation of target protein sequences

Predefined inclusion and exclusion criteria were used to select target proteins with biological relevance and vaccine potential. Inclusion criteria [14]: (1) The protein is a key virulence factor of *M. pneumoniae* or *C. pneumoniae*, with validated roles in pathogen adhesion, invasion, intracellular survival or immune escape; (2) The protein is localized to the extracellular space, outer membrane or cytoplasmic membrane of the pathogen, enabling direct recognition by the host immune system; (3) The protein has high sequence conservation among the analyzed geographically diverse strains to reduce the potential influence of antigenic variation; (4) The protein has been reported to induce protective immune responses in preclinical vaccine studies. Exclusion criteria: Proteins with non-specific immune effects, high antigenic variation, or intracellular localization that cannot be effectively presented were excluded. Based on the above criteria, five target proteins were selected for subsequent epitope screening, namely P1 adhesin and P30 adhesin from *M. pneumoniae*, and MOMP, OmcA, and OmcB from *C. pneumoniae*.

Full-length sequences of target proteins were obtained from experimentally validated reference sequences in the UniProt database (https://www.uniprot.org/) and complete reference genomes of geographically diverse strains from the NCBI Genomes database (https://www.ncbi.nlm.nih.gov/home/genomes/) [15, 16]. Homologous sequences of the five targets from different clinical epidemic strains were also downloaded for subsequent conservancy analysis. All target proteins underwent multi-round validation as follows: (1) Antigenicity: Predicted using the VaxiJen v2.0 server (https://ddg-pharmfac.net/vaxijen/VaxiJen/VaxiJen.html) with a threshold of antigenicity score > 0.4, a widely accepted standard in immunoinformatics vaccine design. This cutoff was selected because it is the recommended threshold for bacterial antigen prediction in VaxiJen and has been widely used in recent immunoinformatics vaccine-design studies [17, 18]; (2) Toxicity and allergenicity: Toxic domains were predicted and excluded using the ToxinPred v2.0 server (https://webs.iiitd.edu.in/raghava/toxinpred2/batch.html), and allergenic proteins were excluded using the AllerTOP v2.1 server (https://www.ddg-pharmfac.net/allertop_test/) [19, 20]; (3) Subcellular localization: Predicted using the DeepLocPro-1.0 server (https://services.healthtech.dtu.dk/services/DeepLocPro-1.0/) [21], only proteins with extracellular, outer membrane or cytoplasmic membrane localization were retained.

### Epitope prediction and screening

#### Prediction and screening of T-cell epitopes

T-cell epitopes were predicted using dedicated tools from the Immune Epitope Database (IEDB) platform. Specifically, CTL epitopes were identified using the IEDB MHC Class I Prediction Tools (https://nextgen-tools.iedb.org/pipeline?tool=tc1), and HTL epitopes were predicted using the IEDB MHC Class II Prediction Tools (https://nextgen-tools.iedb.org/pipeline?tool=tc2) [22].

For CTL epitope prediction, we used the predefined Human Leukocyte Antigen (HLA) class I reference panel of 27 alleles available in the IEDB MHC Class I Prediction Tool. This reference set has been described previously and was designed to provide broad global population coverage (Supplementary Table S1) [22]. The peptide length was set to the canonical 9 amino acids, and binding affinity was analyzed using the NetMHCpan 4.1 EL algorithm. Peptides with a percentile rank < 0.5% were defined as strong binders, following the IEDB/NetMHCpan recommendation that lower percentile ranks indicate stronger predicted HLA binding and are commonly used to prioritize high-confidence CTL epitopes. We further evaluated intracellular processing potential (retaining epitopes with processing score > 0) and immunogenicity (retaining epitopes with positive immunogenicity score > 0) using the built-in tools of the IEDB platform [23].

Candidate CTL epitopes then underwent antigenicity and safety profiling using the same thresholds as protein-level screening: antigenicity score > 0.4 (VaxiJen v2.0), allergenicity score < 0.4 (AlgPred v2.0 server, https://webs.iiitd.edu.in/raghava/algpred2/batch.html) [24], and toxicity score < 0.38 (ToxinPred v3.0 server, https://webs.iiitd.edu.in/raghava/toxinpred3/prediction.php) [25]. Only antigenic, non-allergenic, and non-toxic epitopes were retained.

For HTL epitope prediction, we used the NetMHCIIpan 4.1 EL algorithm with a peptide length of 15 amino acids. The predefined human HLA class II reference panel of 27 alleles available in the IEDB MHC Class II Prediction Tool was used to ensure broad population coverage (Supplementary Table S1) [22]. Peptides with an EL percentile rank < 2% were defined as strong binders, consistent with commonly used IEDB MHC-II epitope screening criteriaforprioritizing high-affinity helper T-cell epitopes[23].

Finally, we evaluated the cytokine induction potential of HTL epitopes to identify epitopes that promote a balanced Th1/Th2 immune response. Interferon-γ (IFN-γ) induction was predicted using the IFNepitope server (https://webs.iiitd.edu.in/raghava/ifnepitope/) [26], interleukin-10 (IL-10) using the IL-10pred server (https://webs.iiitd.edu.in/raghava/il10pred/predict3.php) [27], and interleukin-4 (IL-4) using the IL4pred server (https://webs.iiitd.edu.in/raghava/il4pred/predict.php) [28]. Only HTL epitopes predicted to simultaneously induce all three cytokines were retained.

#### Prediction and screening of linear B-cell epitopes

ABCpred (https://webs.iiitd.edu.in/raghava/abcpred/) [29] was used for the primary prediction of 16-mer linear B-cell epitopes with a threshold of 0.51. To provide complementary validation for the selected epitopes, the corresponding antigenic proteins were further analyzed using BepiPred-3.0 (https://services.healthtech.dtu.dk/services/BepiPred-3.0/) [30]. Since BepiPred-3.0 provides residue-level epitope propensity scores and linear epitope candidate regions rather than fixed-length peptide predictions, the ABCpred-derived LBL epitopes were mapped onto the BepiPred-3.0 output to evaluate whether they overlapped with high-scoring linear B-cell-prone regions. Thus, BepiPred-3.0 was used as a supportive validation tool rather than as a de novo replacement for the original ABCpred-based screening strategy. The retained epitopes were subsequently subjected to the same antigenicity, allergenicity, and toxicity screening as the T-cell epitopes.

### Epitope conservancy analysis

We performed conservancy analysis using the IEDB Epitope Conservancy Analysis Tool (http://tools.iedb.org/conservancy/) to evaluate the predicted conservancy of the selected epitopes across the analyzed geographically diverse strains [31].

Whole-genome sequences of *M. pneumoniae* and *C. pneumoniae* clinical strains from Asia, Europe, Africa, and the Americas were downloaded from the NCBI Genome Database, and homologous sequences of the five target proteins were screened to build protein-specific conservancy analysis libraries. The conservancy level of each epitope was defined as the percentage of homologous sequences containing the identical epitope sequence. A ≥ 90% cutoff was used to balance broad strain coverage with the retention of sufficient immunodominant epitopes for construct design. This threshold has been applied in recent multi-epitope vaccine studies and was selected to reduce the potential influence of antigenic variation across analyzed clinical strains [31].

### Molecular docking of peptides to Human Leukocyte Antigen alleles

Crystal structures of target HLA molecules were obtained from the RCSB Protein Data Bank (https://www.rcsb.org/) [32]. For HLA molecules without available crystal structures, homology models were constructed using the SWISS-MODEL server (https://swissmodel.expasy.org/) [33], with amino acid sequences retrieved from the IPD-IMGT/HLA database (https://www.ebi.ac.uk/ipd/imgt/hla/alleles/) [34].

Molecular docking between T-cell epitopes and their corresponding HLA molecules was performed using the HPEPDOCK2.0 server (http://huanglab.phys.hust.edu.cn/hpepdock/) [35]. Docking results were visualized and analyzed using PyMOL v3.1 software, and interaction characteristics between epitopes and HLA molecules were systematically analyzed using the LigPlot+ tool [36].

### Vaccine sequence design and characterization

The core vaccine constructs were assembled in the following order: N-terminal module, HTL epitope module, CTL epitope module, and LBL epitope module. This modular arrangement was based on published multi-epitope vaccine designs [18]: (1) the β-defensin-3 module in MCV1 was placed at the N terminus to support its predicted immunostimulatory role; (2) the P2 helper epitope in MCV2 and the downstream HTL epitopes were positioned upstream of the CTL and B-cell modules to support CD4⁺ T-cell help; and (3) modular separation of the different epitope classes was intended to reduce junctional interference and preserve independent antigen processing. For structural evaluation and optional recombinant-protein validation, a C-terminal 6×His tag was appended to the protein construct; however, this tag was not included in the final in vivo mRNA vaccine antigen sequence [37].

Different linkers were selected to minimize conformational interference between adjacent modules, with published functional support: (1) the rigid EAAAK linker was used to connect the immunostimulatory module and the HTL epitope module, forming a stable α-helix to separate functionally independent regions [38]; (2) the flexible GPGPG linker was used to connect individual HTL epitopes, helping maintain independent epitope conformation and reduce neo-epitope formation; (3) the short alanine-rich AAA linker was used to connect individual CTL epitopes, serving as a minimal spacer to maintain epitope separation and support efficient processing of the concatenated construct [39]; and (4) the KK linker was used to connect individual LBL epitopes, helping separate adjacent LBL epitopes and reduce junctional interference during construct assembly and antigen processing [40].

Two candidate vaccines (MCV1 and MCV2) were constructed using different N-terminal modules: β-defensin-3 (sequence: GIINTLQKYYCRVRGGRCAVLSCLPKEEQIGKCSTRGRKCCRRK) and the tetanus toxin-derived helper epitope P2 (sequence: QYIKANSKFIGITEL), respectively. β-defensin-3 was selected because it has been associated with Toll-like receptor (TLR) 1/2-mediated innate immune activation and may enhance APC maturation and antigen presentation [41]. In contrast, P2 was included primarily as a universal helper T-cell epitope with promiscuous MHC class II binding potential, and was expected to support CD4⁺ T-cell help rather than direct innate receptor activation [42].

### Multi-dimensional property prediction and screening of the vaccine

Core physicochemical parameters of the vaccine constructs, including amino acid residue count, theoretical isoelectric point, instability index, aliphatic index, in vitro half-life, and grand average of hydropathicity (GRAVY), were calculated using the ProtParam server (https://web.expasy.org/protparam/) [43]. Vaccine antigenicity was evaluated via VaxiJen v2.0, with a qualifying threshold of > 0.4. Allergenicity and toxicity were predicted using the AllerTOP v2.1 and ToxinPred v2.0 servers, respectively; only non-allergenic and non-toxic candidates were retained.

To prevent structural impairment from non-specific cleavage, SignalP 6.0 (https://services.healthtech.dtu.dk/services/SignalP-6.0/) [44] was used to predict endogenous signal peptides and cleavage sites in the core vaccine antigen sequence before the addition of exogenous functional elements. Only candidates lacking abnormal endogenous signal peptides or cleavage sites were retained. Transmembrane topology was analyzed using DeepTMHMM (https://dtu.biolib.com/DeepTMHMM) [45], and only sequences without transmembrane helices were retained. In vitro solubility was predicted using the Protein-Sol server (https://protein-sol.manchester.ac.uk/) [46], with a qualifying threshold of predicted solubility > 0.45.

### Secondary and tertiary structure prediction, optimization, and validation

The secondary structure of the vaccine constructs was predicted using the PSIPRED 4.0 server (http://bioinf.cs.ucl.ac.uk/psipred/) [47], a widely validated tool for secondary structure prediction of multi-epitope vaccine proteins.

For tertiary structure modeling, three protein structure prediction servers were employed: Protenix server (https://protenix-server.com/) [48], AlphaFold 3 server (https://alphafoldserver.com/) [49], and Chai-1 server (https://lab.chaidiscovery.com/) [50]. For each vaccine construct, 5 independent models were generated by each server, yielding a total of 15 initial tertiary structure models. All initial models underwent structure optimization via the structure refinement module of the GalaxyWEB server (https://galaxy.seoklab.org/) [51] to improve conformational rationality and reduce local energy anomalies.

We applied a multi-dimensional hierarchical screening strategy to select the optimal final model, with sequential pre-specified criteria executed in the following order: (1) Ramachandran Plot Validation: Models were first screened using PROCHECK integrated in the SAVES v6.1 server (https://saves.mbi.ucla.edu/) [52], with a pre-set qualifying threshold of ≥ 90% of amino acid residues located in favorable conformational regions. Only models meeting this threshold were advanced to subsequent screening; (2) ERRAT Score Analysis: Models with an ERRAT score ≥ 85 were preferentially retained for further evaluation; (3) ProSA-web Z-score Validation: The structural quality of remaining models was assessed using the ProSA-web server (https://prosa.services.came.sbg.ac.at/prosa.php) [53]. Models with a Z-score within the range of -10 to 0 (consistent with native globular proteins of equivalent size) were considered eligible for final selection; (4) Integrated Model Selection: Among models meeting or approaching the predefined quality criteria, the final model was selected based on the overall balance of the Ramachandran, ERRAT, Verify3D, and ProSA-web assessments.

For one candidate model with an ERRAT score slightly below this threshold (80–85), supplementary validation was performed because the low-scoring regions were confined to linker segments rather than core functional domains. Ramachandran plot analysis showed that ≥ 90% of residues were located in favored regions, and the ProSA-web Z-score remained within the expected range for native proteins. This model was therefore considered acceptable for downstream analysis.

### Disulfide bond engineering

Disulfide bond engineering was performed to enhance the folding efficiency and structural stability of the vaccine constructs, using the Disulfide by Design v2.12 web server (http://cptweb.cpt.wayne.edu/DbD2/) [54].

During analysis, default server parameters were used for the Cα-Cβ-Sγ angle, with the χ3 angle restricted to the range of − 87° to + 97°. A stringent energy threshold of ≤ 2 kcal/mol was set for candidate disulfide bond sites [55]. To preserve the integrity of immune epitopes, residue pairs located within the core sequences of CTL, HTL, or LBL epitopes were strictly excluded; only residue pairs in linker regions or non-functional terminal regions were retained.

Site-directed mutagenesis to cysteine residues was performed for selected candidate residue pairs, and the structural quality of the mutated protein was comprehensively validated using the ProSA-web tool to ensure it met high-quality model criteria. Selected residue pairs were computationally mutated to cysteine to generate exploratory disulfide-engineered structural variants. These mutations were evaluated only at the structural-model level and were not incorporated into the final mRNA-encoded vaccine sequences.

### Conformational B-cell epitope screening

Conformational B-cell epitopes in the vaccine constructs were predicted using the ElliPro web tool from the IEDB resource (http://tools.iedb.org/ellipro/). This prediction is based on the modified Thornton’s method, which integrates residue clustering algorithms, protrusion index, neighboring residue clustering characteristics, and protein spatial conformation [56]. Default parameters were used: maximum distance of 6 Å and minimum score of 0.5.

### Molecular docking of vaccine constructs to immune receptors

β-defensin-3, the N-terminal module of MCV1, has been reported to exert adjuvant-like effects through TLR2-related signaling, which may enhance antigen-presenting cell maturation. Because HLA-mediated antigen presentation is essential for adaptive immune induction, docking was performed between the optimized vaccine constructs and selected immune receptors, including TLR2, HLA-A*02:01, and HLA-DRB1*01:01. TLR4 was included only for an exploratory supplementary analysis. Accordingly, TLR2 docking was considered mainly relevant to MCV1, whereas HLA docking was used to assess the antigen-presentation potential of both constructs.

High-resolution crystal structures of target proteins were obtained from the RCSB PDB database: TLR2 (PDB ID: 6NIG), TLR4 (PDB ID: 8WO1), HLA-A*02:01 (PDB ID: 1HHK), and HLA-DRB1*01:01 (PDB ID: 1AQD). Structures were preprocessed by removing crystal water, small-molecule ligands, and redundant chains, and adding hydrogen atoms and missing side chains.

Initial docking was performed using the ClusPro 2.0 server (https://cluspro.org/help.php), and optimal conformations were selected by ranking via docking cluster score [57]. The top-ranked docking result for each system was submitted to the HADDOCK 2.4 server (https://rascar.science.uu.nl/haddock2.4/) for complex refinement using default energy minimization parameters, with final results evaluated by HADDOCK score [58]. To avoid relying on a single docking pose, HADDOCK refinement results were summarized at the cluster level. For the selected top-ranked cluster of each vaccine–receptor complex, cluster size, HADDOCK score, RMSD from the overall lowest-energy structure, van der Waals energy, electrostatic energy, desolvation energy, restraint violation energy, buried surface area, and Z-score were extracted as available. These cluster-level statistics were summarized descriptively and were not interpreted as experimentally measured binding affinities. Key binding sites and interaction types were analyzed using the PDBsum server (https://www.ebi.ac.uk/thornton-srv/databases/pdbsum/) [59]. HADDOCK scores were interpreted descriptively within the corresponding docking systems and were not used for formal cross-receptor or cross-construct ranking.

### Normal-mode analysis

Normal-mode analysis (NMA) was performed in internal coordinates using the iMODS simulation server (https://imods.chaconlab.org/) to elucidate the collective motion characteristics of protein complexes [60]. Key MD parameters, including eigenvalues, deformability, B-factors, and covariance, were predicted. Eigenvalues were used for quantitative evaluation of motif rigidity, and the deformation characteristics of the protein main chain were predicted.

### Molecular dynamics simulation

MD simulations were performed using GROMACS 2023.3 software (https://www.gromacs.org) to evaluate the predicted dynamic behavior of the selected vaccine–TLR complexes under simulated physiological conditions. The MCV1–TLR2 complex was selected as the biologically relevant primary TLR complex, whereas the MCV2–TLR4 complex was analyzed as an exploratory comparative complex [61].

The AMBER14SB force field was used for parameterization of the vaccine–TLR protein complexes [62]. Each system was solvated with 0.145 mol/L NaCl solution to mimic physiological ionic strength and achieve charge neutralization. Energy minimization was performed using the steepest descent method until the convergence criterion of maximum force ≤ 1000.0 kJ/mol/nm was reached. Equilibration was then performed sequentially using a 500 ps canonical ensemble (NVT) simulation to stabilize the system temperature at 310 K, followed by a 500 ps isothermal-isobaric ensemble (NPT) simulation to stabilize the pressure at 1 atm. A 100 ns production MD simulation was subsequently performed under NPT conditions. The LINCS algorithm was used to constrain covalent bonds involving hydrogen atoms, and the Particle-Mesh Ewald method was used for long-range electrostatic interactions.

To assess reproducibility, two additional independent 100 ns MD simulations were performed for each selected complex in addition to the original production run, resulting in three replicate trajectories per complex. RMSD, radius of gyration (Rg), intermolecular hydrogen-bond counts, and RMSF were analyzed for each replicate trajectory. Quantitative convergence statistics were calculated from the final 20 ns window (80–100 ns), including RMSD mean ± SD, RMSD slope estimated by linear regression of RMSD against simulation time, Rg mean ± SD, and hydrogen-bond count mean ± SD. Across-replicate summaries were calculated from replicate-level mean values. RMSF profiles were summarized as residue-wise mean ± SD across the three replicate trajectories, and chain-level RMSF statistics were further calculated to support residue-fluctuation analysis [63].

### MM-PBSA binding free energy calculation

Binding free energy was calculated using the gmx_MMPBSA v1.61 toolkit to estimate the predicted binding free-energy components of the vaccine–TLR complexes [64]. The MM-PBSA method integrates molecular mechanical energy terms and solvation free-energy components, including van der Waals, electrostatic, polar solvation, and nonpolar solvation contributions. Entropy contributions were not included because of their relatively high estimation uncertainty; therefore, the reported MM-PBSA values represent enthalpy-dominated computational estimates.

To avoid relying on a single conformation or a single MD trajectory, MM-PBSA calculations were performed for three independent replicate MD trajectories of each selected vaccine–TLR complex. For each replicate trajectory, 201 complex frames were used for MM-PBSA calculation. Replicate-level average, standard deviation (SD), and standard error of the mean (SEM) values were extracted for each energy component. The main MM-PBSA table reports across-replicate mean ± between-replicate SD values calculated from the replicate-level averages of the three independent trajectories.

### In silico immune simulation

 In silico immune simulation was performed using the C-ImmSim server (https://kraken.iac.rm.cnr.it/C-IMMSIM/) to generate modeled humoral and cellular immune-response profiles for the vaccine constructs [65]. Simulation parameters were set as follows: total simulation time of 350 days (1050 steps, 8 h per step); a three-dose immunization regimen (1000 vaccine units per dose) on Day 1, Day 28, and Day 56, consistent with clinical mRNA vaccine booster schedules; all other parameters were set to server defaults validated in published immunoinformatics studies [66]. The inherent limitations of the C-ImmSim simulation were noted: the simulation is based on a simplified mathematical model of the mammalian immune system, which cannot fully replicate the complexity of the in vivo immune microenvironment. Thus, these outputs were interpreted as deterministic modeled profiles rather than evidence of actual in vivo immunogenicity, which requires systematic experimental validation.

To provide comparative benchmarking for the immune simulation results, four published multi-epitope vaccine constructs with available full-length amino acid sequences were selected as reference constructs, including two previously reported *M. pneumoniae* vaccine constructs [10, 67] and two previously reported *C. pneumoniae* vaccine constructs [9, 68]. These reference constructs were re-analyzed using the same C-ImmSim settings as those used for MCV1 and MCV2, including the same simulation duration, three-dose immunization schedule, vaccine dose, and default host parameters. Quantitative outputs, including peak total antibody response, memory B-cell response, helper T-cell response, and representative cytokine peaks such as IFN-γ and IL-2, were extracted from the C-ImmSim output plots and compared across constructs.

### Epitope population coverage prediction

To evaluate the global and regional population coverage of the vaccine epitopes, analysis was performed using the Population Coverage tool from the IEDB database (http://tools.iedb.org/population/) [69]. Analysis was performed for both MHC class I and class II epitopes, using the 27 HLA-I and 27 HLA-II alleles selected in the epitope prediction step. In addition to overall global population coverage, regional coverage was calculated for multiple geographical regions and subregions available in the IEDB Population Coverage tool.

### Multi-epitope mRNA vaccine construct design

The final mRNA vaccine constructs were assembled by fusing a tissue plasminogen activator (tPA) signal peptide to the N terminus of each core vaccine through an EAAAK linker [70], and an HLA-B-derived MHC class I trafficking signal to the C terminus through a second EAAAK linker [71]. The C-terminal 6×His tag used in the core protein constructs for structural analysis and optional recombinant-protein validation was excluded from the final mRNA-encoded antigens.

The finalized fusion-protein sequences were reverse-translated and codon-optimized for *Homo sapiens* using the GenSmart™ Codon Optimization Tool, while preserving the encoded amino acid sequences and excluding internal BamHI and XhoI sites. A Kozak sequence (GCCACC) was inserted immediately upstream of the tPA initiation codon [72]. Human β-globin-derived 5′ UTR and α-globin-derived 3′ UTR sequences were placed upstream and downstream of the optimized CDS, respectively [73, 74], with a TAA stop codon inserted before the 3′ UTR.

RNAfold (http://rna.tbi.univie.ac.at//cgi-bin/RNAWebSuite/RNAfold.cgi) analysis was performed separately for MCV1 and MCV2 using sequences comprising the 5′ UTR, Kozak sequence, optimized CDS, TAA stop codon, and 3′ UTR. The 5′ cap and poly(A) tail were not included. Secondary structures were predicted at 37 °C using default parameters [75], and the MFE structure, ensemble free energy, MFE-structure frequency, ensemble diversity, and centroid structure were recorded. Normalized MFE was calculated as MFE divided by transcript length and multiplied by 100.

### Codon-optimization assessment and in silico IVT-template cloning

The CAI values of the final GenSmart-optimized CDSs were independently calculated using the BiologicsCorp Codon Adaptation Index Calculator, with *Homo sapiens* (Mammalian/HEK) selected as the reference set. Only the translated CDSs were analyzed; untranslated regions, the Kozak sequence, stop codon, restriction sites, and poly(A) tail were excluded. CAI values were interpreted as indicators of agreement with the selected human codon-usage reference rather than as direct evidence of protein-expression efficiency.

GC contents and restriction-site profiles were obtained from the final GenSmart output, and the absence of internal BamHI and XhoI sites was confirmed in both CDSs. These parameters were treated as descriptive sequence-level characteristics rather than universal pass–fail criteria.

For in silico IVT-template construction, the complete DNA inserts containing the 5′ UTR, Kozak sequence, optimized CDS, TAA stop codon, and 3′ UTR were inserted downstream of the T7 promoter in the pET-28a(+) backbone using BamHI and XhoI sites [76]. The plasmids were designed for DNA-template propagation and IVT preparation rather than recombinant antigen expression in *Escherichia coli*; therefore, no additional prokaryotic codon optimization was performed.

### Statistical analysis and reporting of computational outputs

**Fig. 1:**
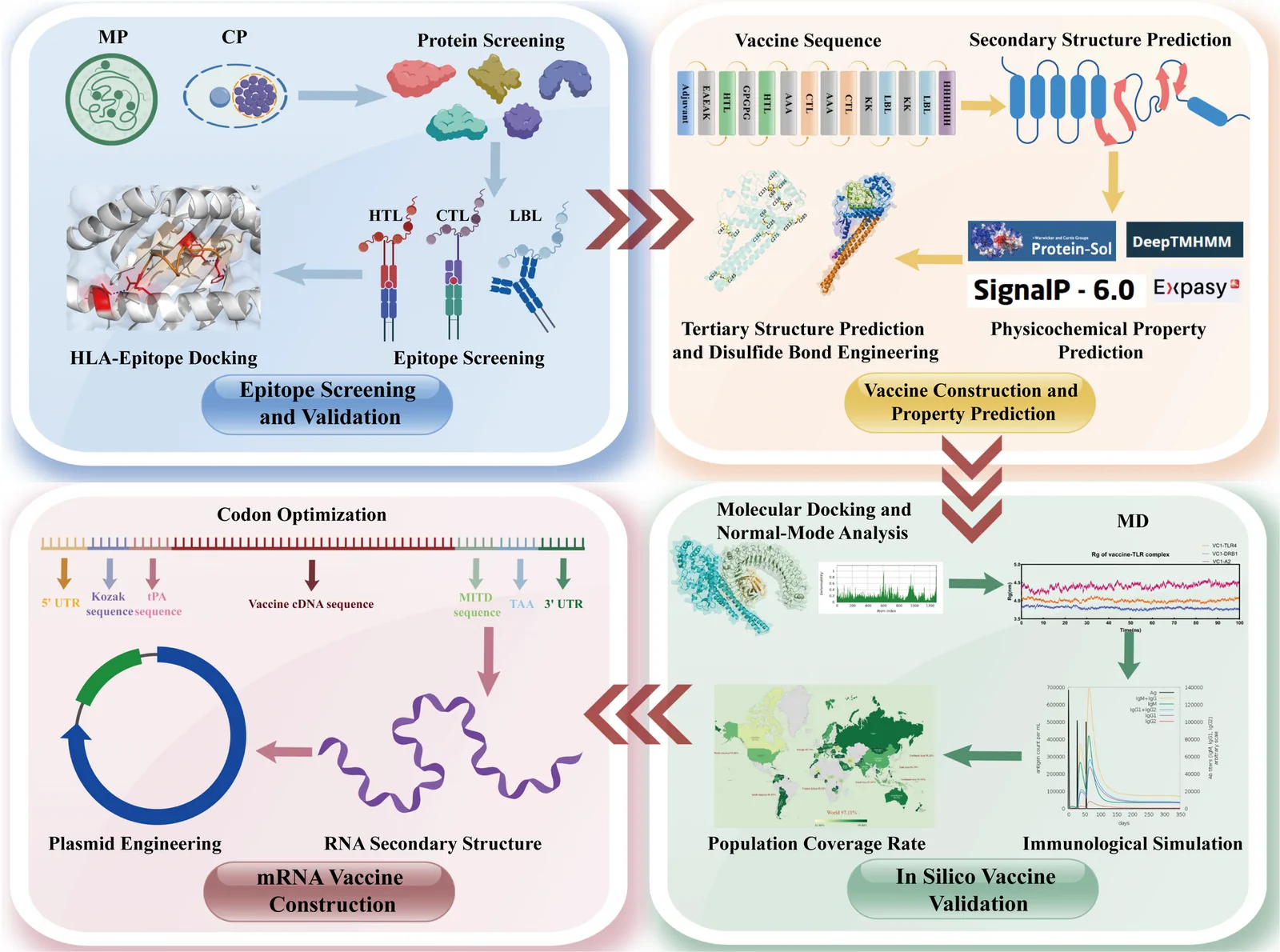
Graphical abstract. (Created with BioGDP.com [78])

Most epitope, structural, docking, population-coverage, codon-optimization, RNA-folding, and single-run C-ImmSim outputs were deterministic and summarized descriptively. HADDOCK results are reported as cluster-level mean ± SD. For MD and MM-PBSA analyses, three independently initialized 100-ns trajectories per complex were treated as computational replicates, with trajectory-level and across-replicate mean ± SD summaries; frames within a trajectory were not considered independent replicates. Formal hypothesis testing between MCV1 and MCV2 was not performed because the constructs were evaluated against different primary TLR systems and only three trajectories were available per system. Accordingly, between-construct differences were interpreted descriptively. Hybrid in silico–in vitro workflows further support distinguishing computational prioritization from subsequent experimental validation [77]. The overall workflow is shown in Fig. 1.

## Results

### Screening of target proteins and epitopes

Five target proteins were selected for epitope screening, including *M. pneumoniae*-derived P1 adhesin (two subtypes: P1-I from strain M129, UniProt ID: P11311; P1-II from strain FH, GenBank accession: GCA_001272835.1) and P30 adhesin (UniProt ID: P75330), as well as *C. pneumoniae*-derived MOMP (UniProt ID: P27455), OmcA (UniProt ID: Q9Z7Z5) and OmcB (UniProt ID: P23700). All candidate proteins met the predefined screening criteria: antigenicity score > 0.4, predicted non-toxic and non-allergenic, and subcellular localization in the extracellular space, outer membrane, or cytoplasmic membrane.

After multi-round screening and verification, a total of 11 CTL epitopes (Table 1), 5 HTL epitopes (Table 2), and 14 LBL epitopes (Table 3) were identified for subsequent vaccine construction. All selected epitopes met the following criteria: (1) antigenicity score > 0.4; (2) no predicted toxicity or allergenicity; (3) strong predicted binding potential with corresponding HLA molecules for CTL and HTL epitopes; (4) predicted potential to induce IL-4, IL-10, and IFN-γ secretion for HTL epitopes; (5) sequence conservancy ≥ 90% among corresponding target strains. These epitopes were prioritized as they simultaneously met all predefined thresholds for antigenicity, safety, HLA binding, cytokine induction, and sequence conservancy.

**Fig. 2:**
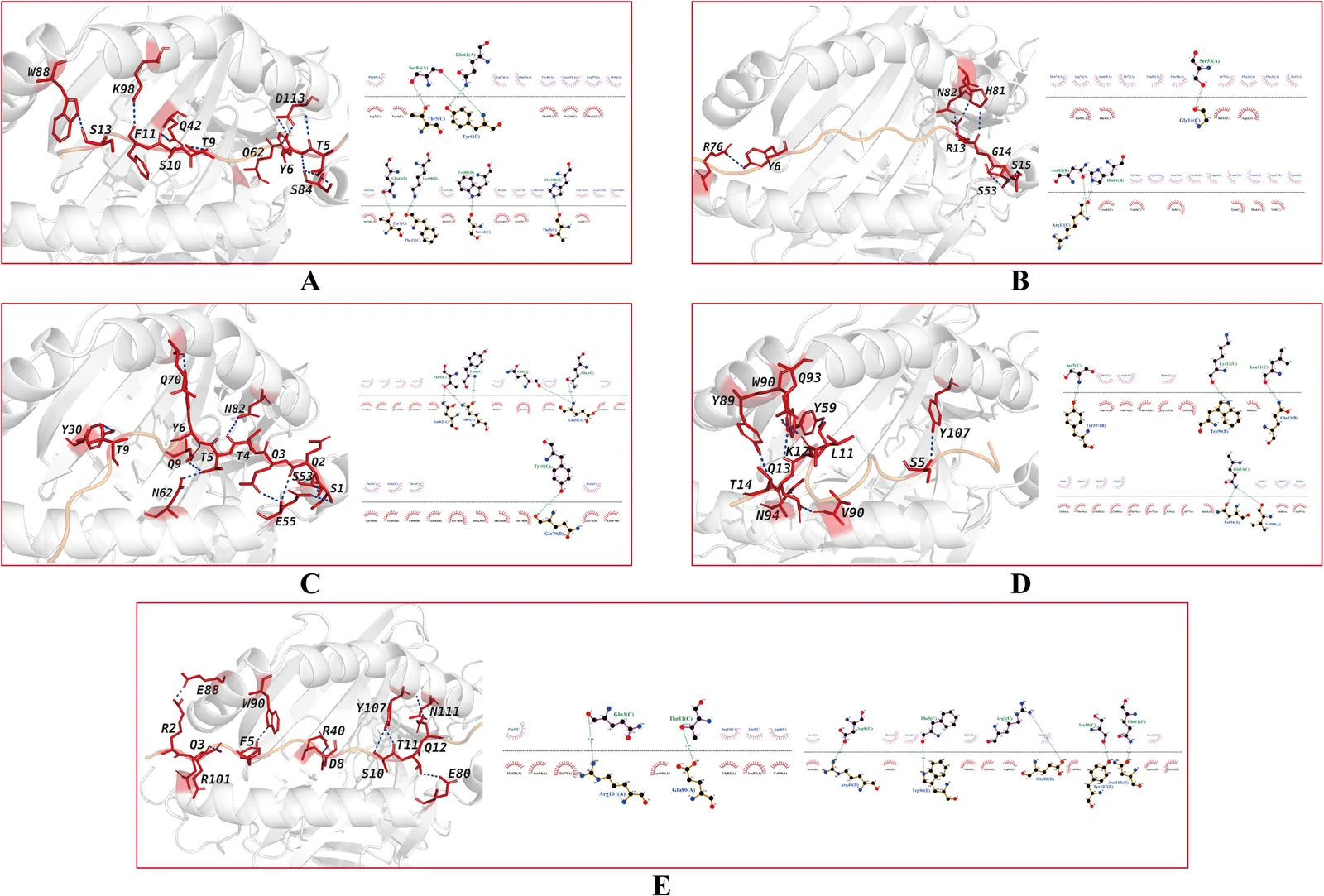
Representative docking conformations and LigPlot+ interaction maps of five selected HTL epitopes with their cognate HLA class II molecules. (**A–E**) Representative peptide–HLA docking models for the five selected HTL epitopes. In each panel, the left subpanel shows the three-dimensional docking conformation, and the right subpanel shows the corresponding LigPlot+ interaction map. Hydrogen bonds and hydrophobic contacts at the peptide–HLA interface are indicated in the interaction diagrams

Molecular docking analysis was performed to evaluate the predicted interaction profiles between selected T-cell epitopes and their cognate HLA molecules. In the resulting docking models, all analyzed T-cell epitopes formed predicted hydrogen bonds and other specific interface interactions with their corresponding HLA molecules. These interactions were visualized using LigPlot+ and PyMOL (Fig. 2; Figs. S1-S2). Supportive validation using BepiPred-3.0 showed that all retained ABCpred-derived LBL epitopes overlapped with high-scoring linear B-cell-prone regions, with 14–16 of 16 residues supported for each epitope (Supplementary Table S2).

**Table 1:** Screen of cytotoxic T lymphocyte epitopes

Protein	Start	Peptide	Allele	EL percentile	Immunogenicity	Antigenicity	Toxicity	Allergenicity	Conservancy
MOMP	29	STAFNLVGL	HLA-A*68:02	0.06	0.1684	0.5467	-	-	100.00% (28/28)
	7	SFSWSVGAR	HLA-A*33:01	0.28	0.09841	1.4287	-	-	100.00% (28/28)
	4	IALNIWDRF	HLA-B*58:01	0.39	0.37696	0.7489	-	-	100.00% (28/28)
OmcA	3	KAVLIAAMF	HLA-B*57:01	0.34	0.09909	1.4131	-	-	100.00% (28/28)
P1(FH)	578	RMAVAGAKF	HLA-B*15:01	0.03	0.03126	0.8369	-	-	100.00% (269/269)
	1383	KMNDDVDGI	HLA-A*02:03	0.22	0.1192	1.1196	-	-	100.00% (269/269)
	525	SIGVFRVRY	HLA-A*30:02	0.21	0.28034	0.7103	-	-	100.00% (269/269)
	543	TGWPYALLF	HLA-A*23:01	0.36	0.07813	0.9601	-	-	100.00% (269/269)
P30	10	KLFLLAWML	HLA-A*32:01	0.26	0.13721	2.3368	-	-	100.00% (273/273)
	19	VLFSALIVL	HLA-A*02:01	0.15	0.03563	0.6658	-	-	100.00% (273/273)
	12	FLLAWMLVL	HLA-A*02:01	0.36	0.10093	2.9915	-	-	100.00% (273/273)

**Table 2:** Screen of helper T lymphocyte epitopes

Protein	Peptide	Start	Allele	EL score	EL percentile	IFN-γ	IL-4	IL-10	Toxicity	Allergenicity	Conservancy
OmcB	GENTVYRICVTNRGS	456	HLA-DRB1*04:01	0.838805	0.44	+	+	+	-	-	100.00% (28/28)
P1(FH)	RTGISQAPKRLKQTS	1591	HLA-DRB1*13:02	0.533285	1.7	+	+	+	-	-	100.00% (269/269)
P1(M129)	LTPWTYRNTSFSSLP	66	HLA-DPA1*02:01/DPB1*01:01	0.276806	0.3	+	+	+	-	-	100.00% (269/269)
	LRQSFGTDHSTQPQP	1271	HLA-DRB3*01:01	0.382791	1.4	+	+	+	-	-	100.00% (269/269)
	SQQTTYDPTRTEAAL	106	HLA-DRB1*04:01	0.612019	1.9	+	+	+	-	-	100.00% (269/269)

**Table 3:** Screen of linear B lymphocyte epitopes

Protein	Rank	Sequence	Start	Score	Antigenicity	Toxicity	Allergenicity	Conservancy
MOMP	9	VDADKWSLTAEARLIN	418	0.83	0.9855	-	-	100.00% (28/28)
	10	YHEWQVGASLSYRLNS	321	0.82	1.1807	-	-	100.00% (28/28)
OmcA	7	AVLIAAMFCGVVSLSS	79	0.82	0.4030	-	-	100.00% (28/28)
OmcB	9	RVLSFNLGDMRPGDKK	343	0.85	1.2960	-	-	100.00% (28/28)
	28	SSDTLTSPVSDTENTH	612	0.64	0.6522	-	-	100.00% (28/28)
P1(FH)	1	MWAWQVYNWSNKLTDQ	735	0.95	0.9981	-	-	100.00% (269/269)
P1(M129)	6	GGSQTMITGGSPRRTL	934	0.9	1.3087	-	-	100.00% (269/269)
	11	VGVGRLSESNAAKMND	1428	0.85	0.7338	-	-	100.00% (269/269)
	12	ITMGDTATVPRLLYDE	656	0.84	0.6694	-	-	100.00% (269/269)
	13	GSPRRTLDQANLQLWT	943	0.83	0.7596	-	-	100.00% (269/269)
P30	3	PPRPGMPPQPGFPPKR	318	0.9	0.5651	-	-	100.00% (273/273)
	4	PRPGMQPPRPGMPPQP	312	0.89	0.8252	-	-	100.00% (273/273)
	16	FLLAWMLVLFSALIVL	71	0.68	1.7320	-	-	100.00% (273/273)
	17	LILVQHNNTELTEVKS	89	0.62	0.6049	-	-	100.00% (273/273)

### Vaccine design and key property evaluation

**Fig. 3:**
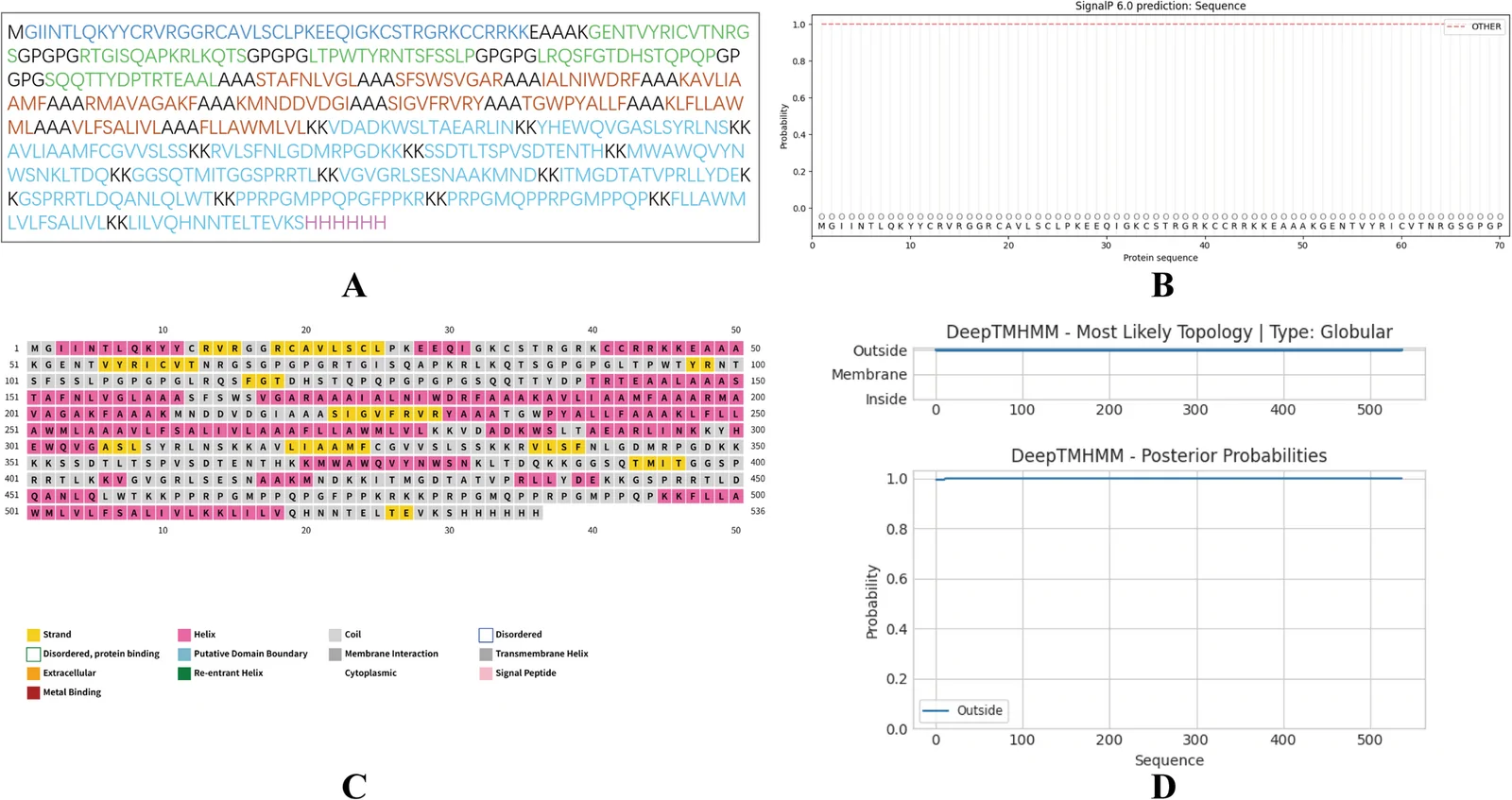
Sequence organization and basic structural characterization of the MCV1 core protein construct. **A** Schematic organization of the MCV1 core protein construct, including the β-defensin-3 immunostimulatory module, linkers, HTL epitopes, CTL epitopes, LBL epitopes, and the C-terminal 6×His tag used for structural evaluation; **B** SignalP 6.0 prediction profile; **C** predicted secondary-structure composition; **D** DeepTMHMM topology prediction

Two candidate multi-epitope vaccine constructs were generated, designated MCV1, containing a β-defensin-3 immunostimulatory module, and MCV2, containing the tetanus toxin-derived P2 helper-epitope module. The core protein constructs used for structural evaluation comprised the respective N-terminal module, 11 CTL epitopes, 5 HTL epitopes, 14 LBL epitopes, the selected linkers, and a C-terminal 6×His tag (Fig. 3A; Fig. S3A). The 6×His tag was included for structural evaluation and potential recombinant-protein purification or validation but was excluded from the final mRNA-encoded antigen sequences.

The antigenicity scores of MCV1 and MCV2 were 0.7978 and 0.7780, respectively, both exceeding the predefined threshold of 0.4. No potential toxicity or allergenicity was predicted for either core protein construct. Before the addition of the exogenous tPA signal peptide and HLA-B-derived trafficking signal, neither core construct contained an abnormal endogenous signal peptide or predicted transmembrane helix (Fig. 3B, D, Fig. S3B, D).

Key physicochemical properties of the two vaccines were summarized as follows: MCV1 consisted of 536 amino acids, with a molecular weight of 58.3 kDa, predicted isoelectric point of 11.04, instability index of 33.63, aliphatic index of 77.67, and GRAVY of -0.267. MCV2 consisted of 506 amino acids, with a molecular weight of 54.8 kDa, predicted pI of 10.84, instability index of 30.92, aliphatic index of 79.58, and GRAVY of -0.22. Both vaccines met the classification criteria for stable proteins, with predicted in vitro half-lives of 30 h in mammalian reticulocytes, > 20 h in yeast, and > 10 h in *E. coli*. The predicted solubility coefficients of MCV1 and MCV2 were 0.508 and 0.517, respectively, both exceeding the reference threshold of 0.45.

### Prediction and validation of secondary and tertiary structures of vaccines

Secondary structure prediction results showed that MCV1 comprised 52.2% coils, 38.4% helices, and 9.4% strands (Fig. 3C), while MCV2 comprised 54.5% coils, 38.2% helices, and 7.3% strands (Fig. S3C). Both constructs had helix proportions of approximately 40%, indicating broadly similar predicted secondary-structure compositions.

The optimized tertiary-structure models generated by AlphaFold 3, Chai-1, and Protenix showed different profiles across the ERRAT, Verify3D, and ProSA-web metrics (Supplementary Table S3). The AlphaFold 3 models yielded relatively high ERRAT scores but low Verify3D scores (≤ 20.55%), indicating limited compatibility between the three-dimensional structures and their residue-level sequence environments. The Protenix models yielded the highest Verify3D scores and acceptable ProSA-web Z-scores, but their ERRAT scores were below the preferred threshold of 85. In contrast, the Chai-1 models provided the most balanced overall profiles: their ProSA-web Z-scores were within the predefined acceptable range, their Verify3D scores were substantially higher than those of the AlphaFold 3 models, and their ERRAT scores met or were close to the preferred threshold. Accordingly, the refined Chai-1 models were selected for subsequent analyses.

**Fig. 4:**
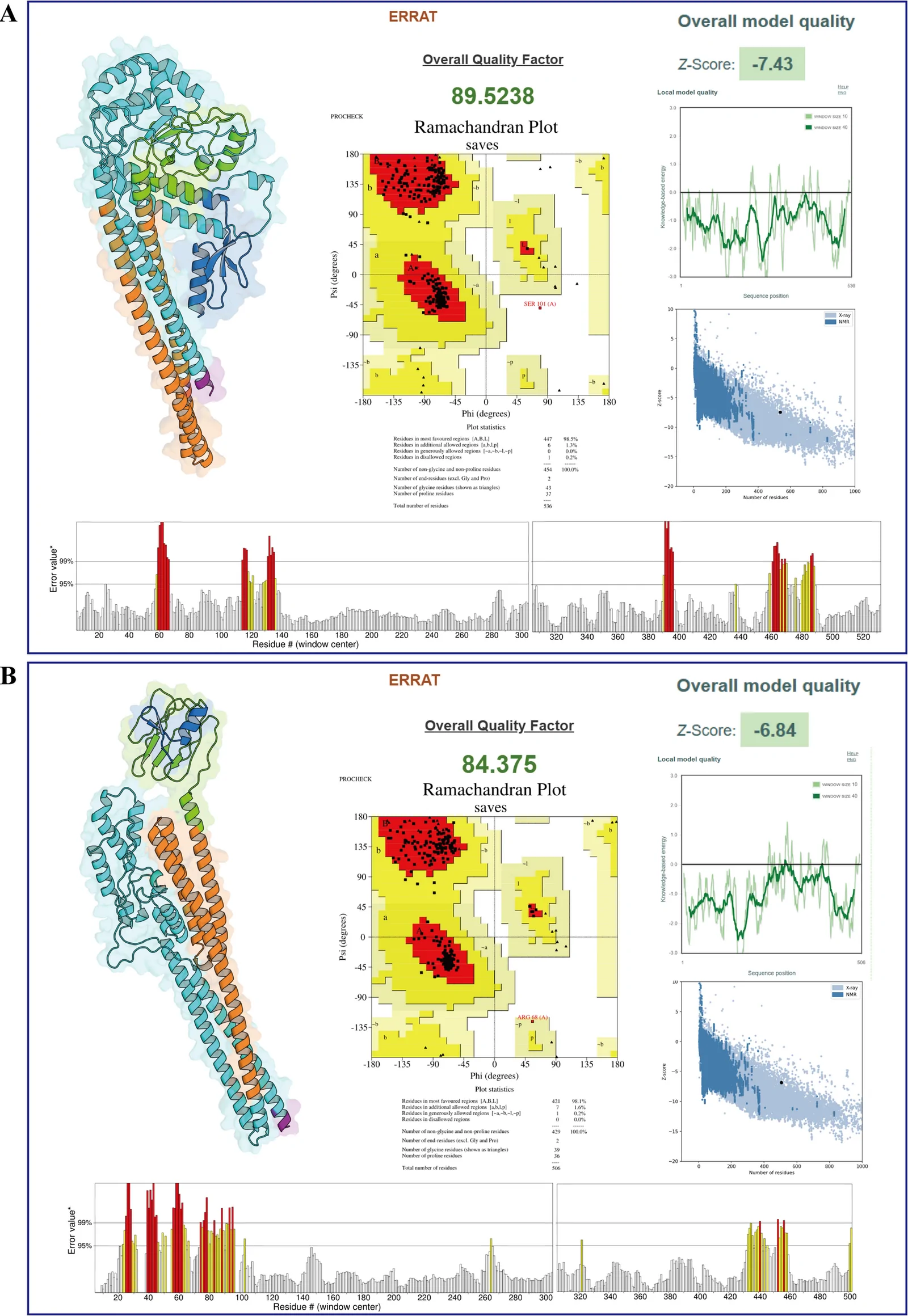
Refined tertiary structures and structural quality assessment of MCV1 and MCV2. **A** Final refined three-dimensional model of MCV1 together with the corresponding Ramachandran plot, ProSA-web Z-score/energy plot, and residue-wise error profile; **B** Final refined three-dimensional model of MCV2 together with the corresponding Ramachandran plot, ProSA-web Z-score/energy plot, and residue-wise error profile

For the final MCV1 model, the ERRAT score was 89.5238 and the ProSA-web Z-score was − 7.43. PROCHECK Ramachandran plot analysis showed that 98.5% of residues were located in the most favored regions and 1.3% in additionally allowed regions, with no residues in disallowed regions (Fig. 4A). For the final MCV2 model, the ERRAT score was 84.375 and the ProSA-web Z-score was − 6.84. Ramachandran plot analysis showed that 98.1% of residues were located in the most favored regions and 1.6% in additionally allowed regions, with no residues in disallowed regions (Fig. 4B). Although the ERRAT score of MCV2 was slightly below the preferred threshold of 85, the model was retained after supplementary validation because its Ramachandran and ProSA-web results were acceptable and the lower-quality regions were mainly confined to linker segments rather than principal functional regions.

### Disulfide bond engineering of vaccines and evaluation

For MCV1, 46 potential residue pairs were initially identified, and 8 pairs met the screening criteria: CYS41-CYS12, CYS60-CYS325, GLY69-GLY131, SER121-THR396, THR136-GLN392, ALA152-MET275, ALA245-THR526, and ASN313-ASP385. For MCV2, 47 potential residue pairs were initially identified, and 5 pairs met the screening criteria: ALA18-LEU82, ALA143-ALA155, TRP149-SER302, PHE176-ALA191, and GLY187-ALA401. Detailed energy scores for each residue pair are provided in Supplementary Table S4.

**Fig. 5:**
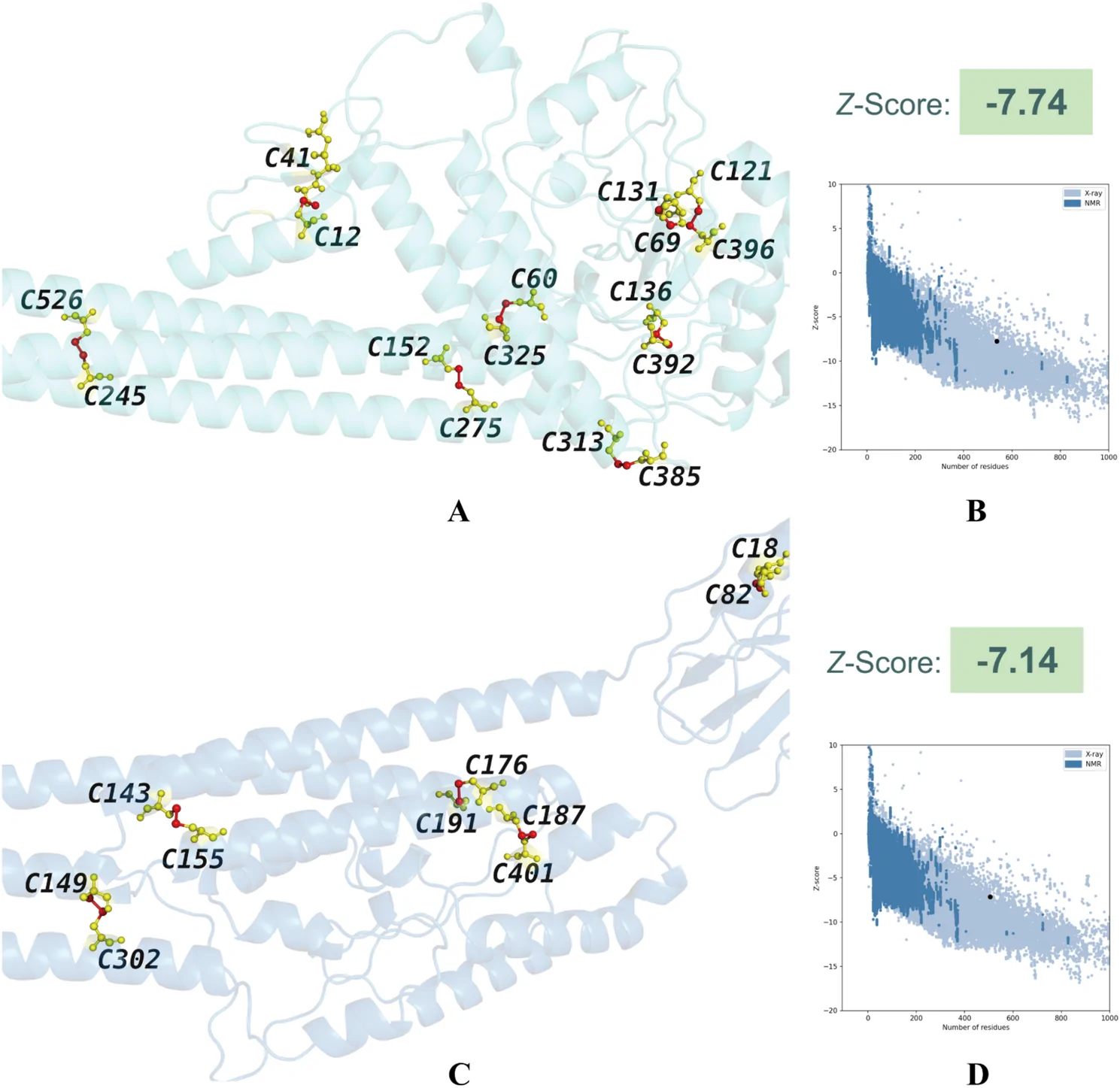
Disulfide bond-engineered models and ProSA-web validation of MCV1 and MCV2. **A**, **C** Three-dimensional models of MCV1 and MCV2 after disulfide bond engineering, with the introduced disulfide bond sites indicated; **B**, **D** Corresponding ProSA-web Z-score plots of the engineered MCV1 and MCV2 models

The three-dimensional structures of the two vaccine constructs after disulfide bond introduction are shown in Fig. 5A and C. In the exploratory disulfide-engineered structural variants, the ProSA-web Z-score shifted from − 7.43 to − 7.74 for MCV1 and from − 6.84 to − 7.14 for MCV2 (Fig. 5B and D). These changes indicate altered predicted structural-quality profiles but do not establish improved structural stability.

### Molecular docking

#### TLR docking

**Fig. 6:**
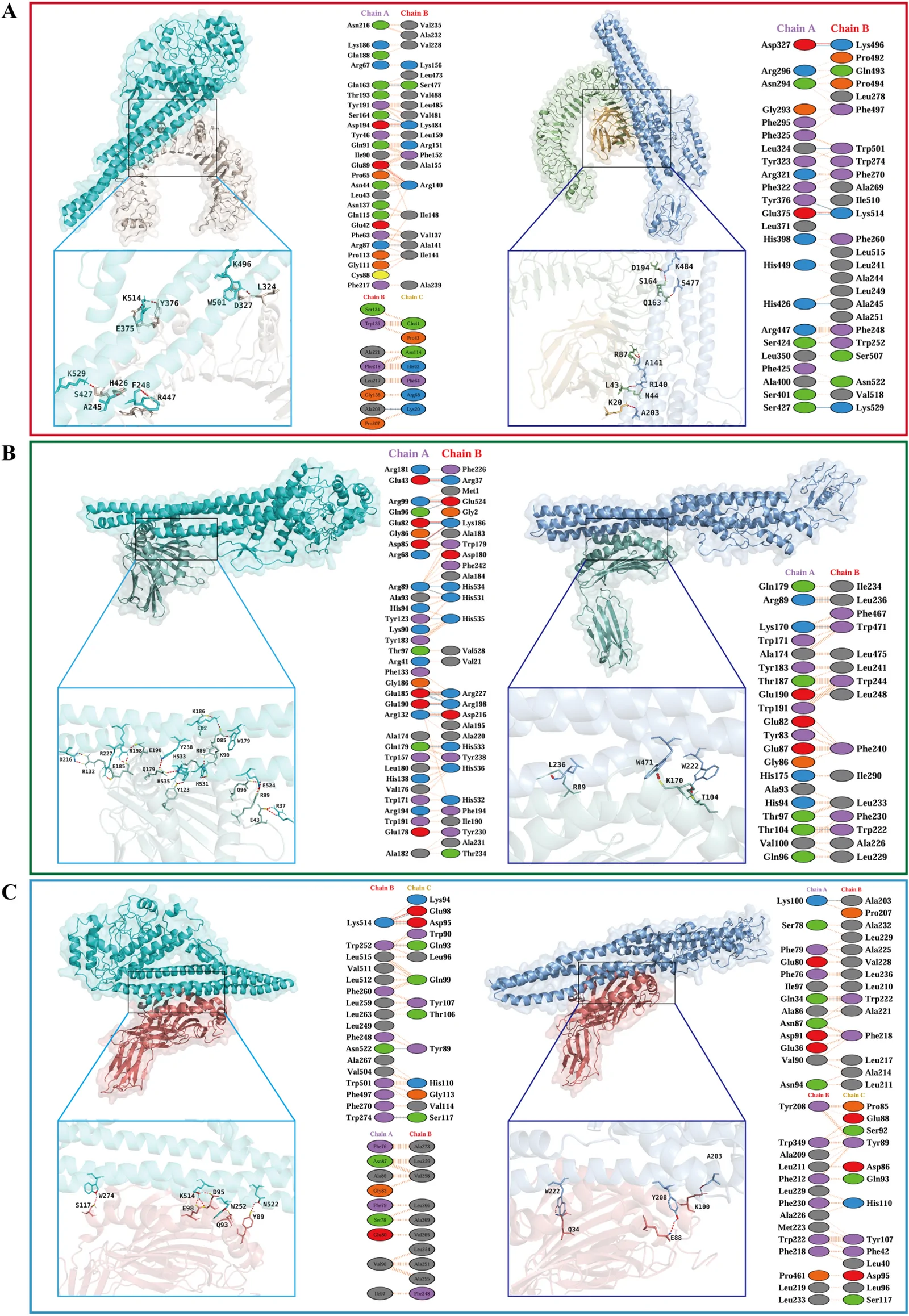
Molecular docking models of the vaccine constructs with selected immune receptors. **A** Docking models of MCV1 with TLR2 (left) and MCV2 with TLR4 (right); **B** Docking models of MCV1 with HLA-A*02:01 (left) and MCV2 with HLA-A*02:01 (right); **C** Docking models of MCV1 with HLA-DRB1*01:01 (left) and MCV2 with HLA-DRB1*01:01 (right). For each subpanel, the upper view shows the overall docking conformation, and the enlarged inset highlights representative interface interactions

Docking between MCV1 and TLR2 was performed as the biologically relevant primary analysis, given the reported association of the β-defensin-3 module with TLR2-related signaling. Docking between MCV2 and TLR4 was performed as an exploratory supplementary analysis. The ClusPro weighted scores for the cluster-center model and the lowest-scoring cluster member were − 1061.3 and − 1326.3 for MCV1–TLR2 and − 1161.9 and − 1315.4 for MCV2–TLR4, respectively. These ClusPro scores were treated as algorithmic docking outputs rather than physical binding energies. After HADDOCK refinement, the selected clusters had HADDOCK scores of − 394.3 ± 7.3 for MCV1–TLR2 and − 399.0 ± 5.5 for MCV2–TLR4, with 20 models in each cluster. The RMSD values from the overall lowest-energy structure were 0.7 ± 0.4 and 0.3 ± 0.2, respectively. Interface analysis identified six predicted hydrogen bonds in each complex. Detailed cluster-level HADDOCK statistics are provided in Supplementary Table S6, and representative docking conformations are shown in Fig. 6A and Fig. S4A, B.

#### HLA docking

Docking was also performed between both vaccine constructs and HLA-A*02:01 and HLA-DRB1*01:01 to evaluate predicted antigen-presentation-related interaction profiles. For MCV1, the ClusPro weighted scores for the cluster-center model and the lowest-scoring cluster member were − 1183.1 and − 1202.3 for MCV1–HLA-A*02:01 and − 1066.4 and − 1253.0 for MCV1–HLA-DRB1*01:01, respectively. After HADDOCK refinement, the corresponding cluster-level HADDOCK scores were − 282.3 ± 1.4 and − 655.9 ± 1.1. For MCV2, the ClusPro weighted scores were − 1128.6 and − 1172.7 for MCV2–HLA-A*02:01 and − 1196.1 and − 1248.9 for MCV2–HLA-DRB1*01:01, respectively. The corresponding HADDOCK scores were − 162.3 ± 1.5 and − 669.0 ± 4.4. All selected HADDOCK clusters contained 20 models, and the RMSD values from the overall lowest-energy structure ranged from 0.2 ± 0.1 to 0.4 ± 0.3. Interface analysis identified 18 and five predicted hydrogen bonds for MCV1 with HLA-A*02:01 and HLA-DRB1*01:01, respectively, and three predicted hydrogen bonds for each corresponding MCV2–HLA complex. Detailed cluster-level statistics are provided in Supplementary Table S6, and representative docking models are shown in Fig. 6B, C and Fig. S4C–F.

### Normal-mode analysis

**Fig. 7:**
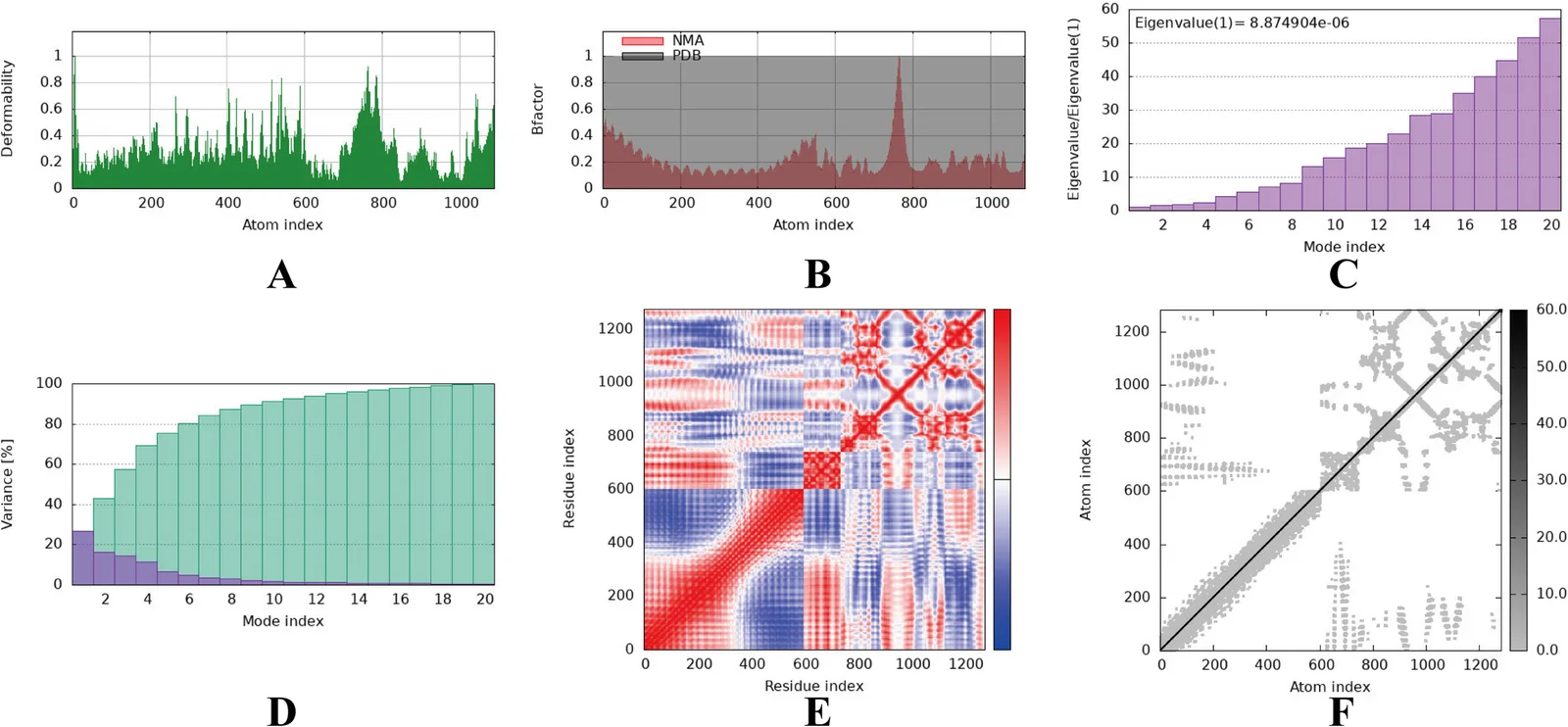
Normal-mode analysis of the MCV1–TLR2 complex. **A** Deformability profile; **B** B-factor profile; **C** Eigenvalue distribution; **D** Variance plot; **E** Covariance matrix; **F** Elastic network model

NMA of the MCV1–TLR2 complex showed generally low deformability across most regions, with several localized peaks indicating limited flexible segments (Fig. 7A). The B-factor profile was similarly low overall, except for a distinct fluctuation peak in one local region (Fig. 7B). The first-mode eigenvalue was 8.874904e-06 (Fig. 7C). The variance plot showed that the major conformational motions were dominated by low-frequency modes, as cumulative variance rose rapidly and approached 100% with increasing mode number (Fig. 7D). The covariance map revealed mainly correlated motions within several residue blocks, together with some anti-correlated motions between distinct regions (Fig. 7E). The elastic network further supported the presence of densely connected rigid regions and relatively flexible local segments (Fig. 7F). Overall, these results indicate that the modeled MCV1–TLR2 complex possesses a relatively stable structural framework with localized conformational flexibility. The corresponding NMA results for the exploratory MCV2–TLR4 complex are shown in Fig. S5.

### Molecular dynamics simulations and MM-PBSA

**Fig. 8:**
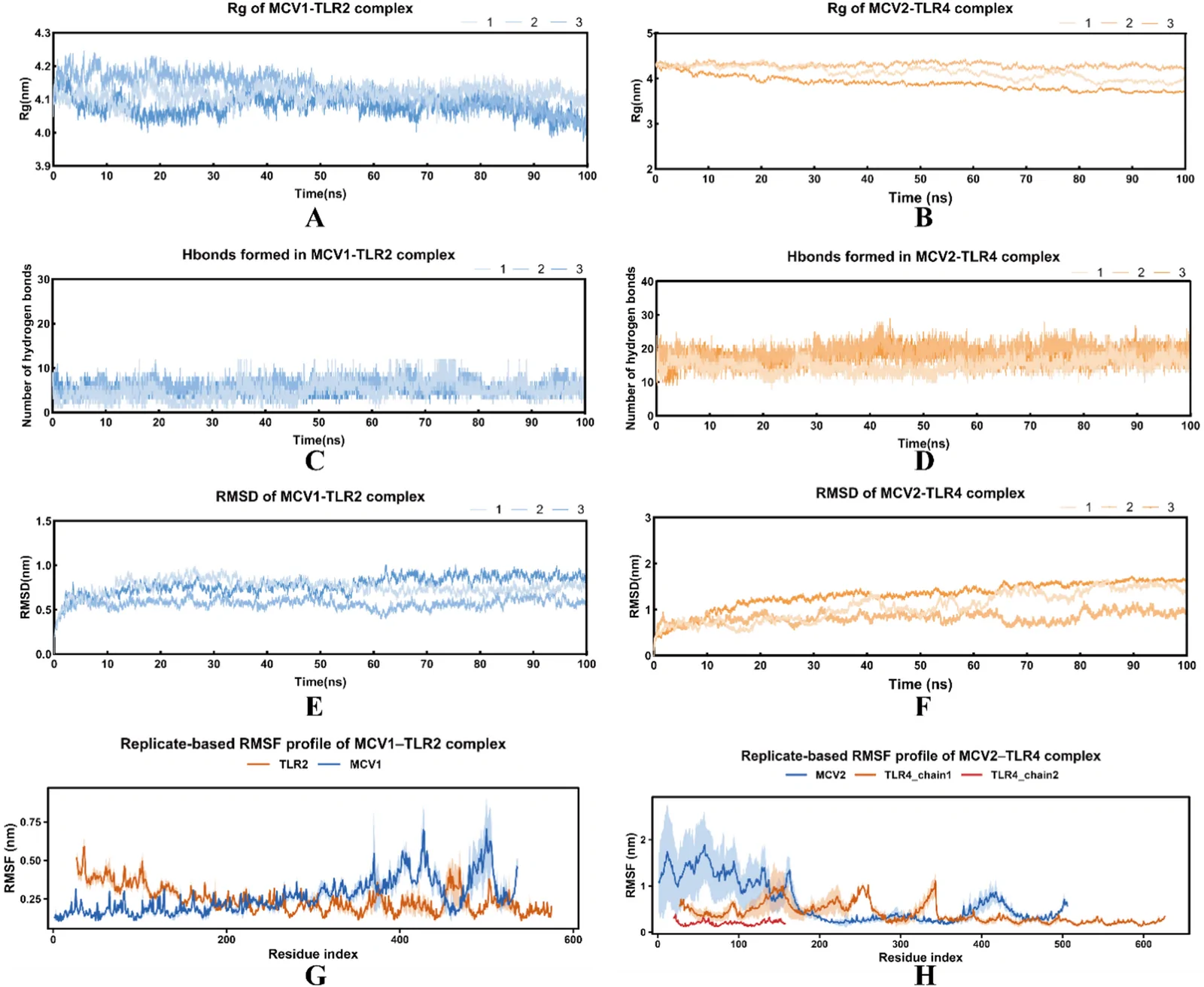
Replicate molecular dynamics simulation profiles of the MCV1–TLR2 and exploratory MCV2–TLR4 complexes over 100 ns. Three independently initialized 100-ns MD trajectories were analyzed for each complex. **A**,** B** Radius-of-gyration trajectories of MCV1–TLR2 and MCV2–TLR4, respectively. **C**,** D** Time-dependent intermolecular hydrogen-bond counts. **E**,** F** RMSD trajectories. **G**,** H** Replicate-based residue-level RMSF profiles, summarized across the three trajectories. Lines 1–3 in panels A–F represent the independent trajectories. All between-complex comparisons are descriptive because the complexes contain different receptor systems

The dynamic behavior of the MCV1–TLR2 and exploratory MCV2–TLR4 complexes was evaluated using three independently initialized 100-ns MD trajectories per complex (Fig. 8). During the final 20-ns window (80–100 ns), the MCV1–TLR2 complex showed relatively consistent replicate trajectories, with an across-replicate RMSD of 0.739 ± 0.136 nm, an Rg of 4.082 ± 0.033 nm, and an intermolecular hydrogen-bond count of 4.07 ± 1.02. The average RMSD slope during this window was close to zero (0.00111 nm/ns), indicating limited late-stage drift under the present simulation settings (Supplementary Table S7).

For the exploratory MCV2–TLR4 complex, the corresponding final-window values were 1.360 ± 0.352 nm for RMSD, 3.972 ± 0.276 nm for Rg, and 15.25 ± 5.70 for the intermolecular hydrogen-bond count. The between-replicate dispersion was numerically larger than that observed for MCV1–TLR2, as reflected by the larger between-replicate SDs of these trajectory-level summaries. Nevertheless, the average RMSD slope was also close to zero (0.00104 nm/ns), and predicted intermolecular hydrogen bonds remained detectable across all three trajectories (Supplementary Table S7). These findings indicate limited average late-window drift but greater trajectory-level heterogeneity in the MCV2–TLR4 system.

Replicate-based RMSF analysis showed different residue-level fluctuation profiles for the two complexes. In the MCV1–TLR2 system, the MCV1 and TLR2 chains had mean RMSF values of 0.262 and 0.245 nm, respectively. In the MCV2–TLR4 system, the MCV2 and TLR4 chain 1 regions showed mean RMSF values of 0.649 and 0.398 nm, respectively, whereas TLR4 chain 2 showed a lower mean RMSF of 0.201 nm (Supplementary Table S8). The larger RMSF values in selected MCV2 and TLR4 chain 1 regions indicate numerically greater local flexibility under the present simulation settings. Because the two complexes contained different receptor systems, these comparisons were interpreted descriptively and were not used to establish the overall superiority or stability of either vaccine construct.

MM-PBSA calculations were subsequently performed using 201 complex frames from each of the three independent trajectories per complex. The main results are reported as across-replicate mean ± between-replicate SD values in Table 4, and the replicate-level energy components are provided in Supplementary Table S9. The total binding free energy (ΔTOTAL) remained negative in all three trajectories for both complexes. The across-replicate ΔTOTAL values were − 125.19 ± 20.13 kcal/mol for MCV1–TLR2 and − 126.87 ± 12.73 kcal/mol for MCV2–TLR4. Van der Waals and nonpolar solvation terms contributed favorably to the calculated binding energetics in both systems, whereas the electrostatic and polar-solvation terms showed substantial compensatory variation, particularly for MCV2–TLR4. These results indicate negative predicted binding free energies under the specified MM-PBSA settings. However, because the complexes involved different receptors and only three independent trajectories were analyzed per system, the values were not subjected to formal head-to-head hypothesis testing or used to rank the two constructs. They should be interpreted as computational energy estimates rather than experimentally measured binding affinities.

**Table 4:** MM-PBSA binding free-energy components averaged across replicate MD trajectories

Complex	ΔVDWAALS: Across-replicate mean ± SD	ΔEEL: Across-replicate mean ± SD	ΔEPB: Across-replicate mean ± SD	ΔENPOLAR: Across-replicate mean ± SD	ΔGGAS: Across-replicate mean ± SD	ΔGSOLV: Across-replicate mean ± SD	ΔTOTAL: Across-replicate mean ± SD
MCV1–TLR2	-140.39 ± 36.02	-1769.68 ± 208.65	1802.00 ± 192.37	-17.12 ± 3.77	-1910.07 ± 200.98	1784.88 ± 193.66	-125.19 ± 20.13
MCV2–TLR4	-151.37 ± 14.27	-912.81 ± 2239.55	955.00 ± 2241.08	-17.69 ± 1.36	-1064.18 ± 2252.35	937.31 ± 2239.91	-126.87 ± 12.73

Values are reported as across-replicate mean ± between-replicate SD (kcal·mol⁻¹) from three independently initialized MD trajectories per complex, with 201 frames analyzed per trajectory; trajectories, rather than individual frames, were treated as replicates. ΔVDWAALS represents van der Waals energy; ΔEEL, electrostatic energy; ΔEPB, polar solvation energy; ΔENPOLAR, nonpolar solvation energy; ΔGGAS, gas-phase free energy; ΔGSOLV, solvation free energy; and ΔTOTAL, total binding free energy. More negative ΔTOTAL values indicate more favorable predicted binding energetics under the MM-PBSA model. These values are computational estimates rather than experimentally measured binding affinities

### Immunization simulation

**Fig. 9:**
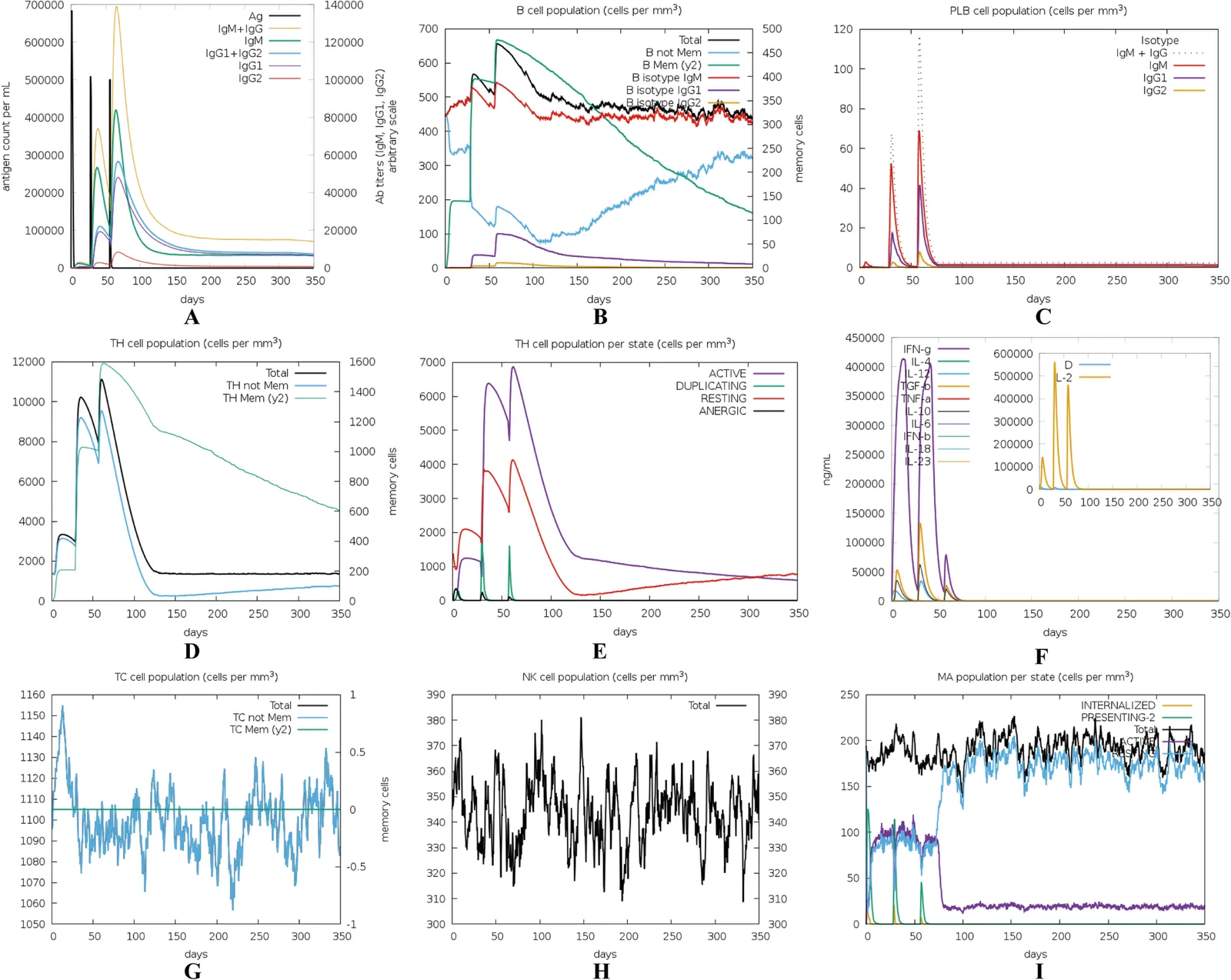
C-ImmSim-predicted post-immunization immune-response profiles for MCV1. **A** Antibody titers; **B** B cell levels; **C** Plasma cell counts; **D** Helper T (Th) cell levels; **E** Subset populations of Th cells; **F** Cytokine secretion levels; **G** Cytotoxic T lymphocyte levels; **H** NK cell counts; **I** Macrophage counts

Both constructs exhibited broadly similar immune-response patterns in the C-ImmSim simulation. Following sequential immunizations, simulated antigen levels declined more rapidly after booster doses, while predicted antibody responses increased progressively and reached their highest levels after the final immunization. For MCV1, the combined simulated antibody response peaked at approximately 700,000 arbitrary units, whereas the corresponding peak for MCV2 was approximately 620,000 (Fig. 9A, Fig. S6A).

The simulation further predicted increases in total B-cell and memory B-cell populations after immunization for both constructs, together with transient rises in plasma B lymphocytes and isotype-associated responses, suggesting simulated patterns consistent with potential humoral immune activation and memory formation in silico (Fig. 9B, C; Fig. S6B, C). Predicted helper T-cell responses showed a similar trend, with higher total TH-cell levels observed after booster immunizations. In addition, shifts among active, duplicating, resting, and anergic TH-cell states were observed in the simulation, suggesting dynamic T-helper-cell state transitions during the modeled immune response (Fig. 9D, E; Fig. S6D, E).

Simulated cytokine profiles also suggested that both constructs may be associated with increased cytokine-related outputs, particularly IFN-γ and IL-2, along with detectable IL-10, TGF-β, and other cytokines, which may be consistent with a Th1-skewed immune pattern in silico (Fig. 9F, Fig. S6F). In contrast, predicted TC-cell responses appeared comparatively modest, whereas NK-cell populations remained relatively stable with only minor fluctuations over time in the simulation (Fig. 9G, H; Fig. S6G, H). Simulated macrophage populations showed modeled changes in state distribution after immunization, including presenting and internalized states (Fig. 9I, Fig. S6I).

To provide descriptive context for the C-ImmSim outputs, four published multi-epitope vaccine constructs with available full-length amino acid sequences were re-simulated using the same duration, immunization schedule, vaccine dose, and default host parameters as MCV1 and MCV2. The resulting antibody, memory B-cell, helper T-cell, IFN-γ, and IL-2 outputs for both study constructs were within the ranges observed for the reference constructs under these identical simulation settings (Supplementary Table S10).

Descriptive comparison between MCV1 and MCV2 showed that MCV1 had a numerically higher simulated total-antibody peak (~ 7.0 × 10⁵ versus ~ 6.2 × 10⁵). The simulated memory B-cell peaks (~ 4.7 × 10² cells/mm³), helper T-cell peaks (~ 1.1 × 10⁴ cells/mm³), and IFN-γ peaks (~ 4.1 × 10⁵ ng/mL) were broadly similar between the two constructs, whereas the simulated IL-2 peak was slightly higher for MCV2 (~ 5.8 × 10⁵ versus ~ 5.6 × 10⁵ ng/mL). These values were extracted or visually estimated from deterministic C-ImmSim output plots and were not subjected to inferential statistical testing.

Overall, both constructs generated simulated response profiles involving humoral, helper T-cell, cytokine, and innate-cell components under the specified C-ImmSim settings. The benchmarking analysis provides descriptive in silico context but does not demonstrate that either construct is immunologically superior or confirm actual antigen expression, immunogenicity, or protective efficacy.

### Population coverage

As shown in Table 5, the combined global population coverage of the selected epitopes was predicted to be 97.90%, with an average hit of 3.59 and a PC90 value of 1.54. However, marked regional heterogeneity was observed. The highest predicted coverage values were found in North America (99.99%), Europe (99.92%), West Africa (99.65%), Central Africa (99.47%), South Asia (99.34%), and East Africa (99.27%). In contrast, lower predicted coverage was observed in East Asia (78.01%), South Africa (57.71%), and Southeast Asia (57.19%). These results indicate high predicted global population coverage overall, but substantial regional heterogeneity.

**Table 5:** Predicted global and regional population coverage of the selected T-cell epitopes based on combined MHC class I and class II analysis

Population/area	Class combined		
	Coverage	Average_hit	Pc90
World	97.90%	3.59	1.54
North America	99.99%	4.03	1.96
Europe	99.92%	4.11	2.1
West Africa	99.65%	4.06	2.23
Central Africa	99.47%	3.38	1.84
South Asia	99.34%	3.13	1.68
East Africa	99.27%	3.7	2.01
South America	97.99%	2.65	1.31
Northeast Asia	92.34%	2.37	1.08
Oceania	88.50%	1.86	0.87
North Africa	85.41%	2.52	0.69
Southwest Asia	81.56%	2.16	0.54
West Indies	78.79%	2.33	0.47
East Asia	78.01%	1.89	0.45
South Africa	57.71%	1.14	0.24
Southeast Asia	57.19%	1.46	0.23
Average	84.03%	2.62	1.14
Standard deviation	21.86%	1.09	0.72

Population-coverage estimates are deterministic outputs generated by the IEDB Population Coverage tool and are presented descriptively

### Screening of conformational B-cell epitopes

**Fig. 10:**
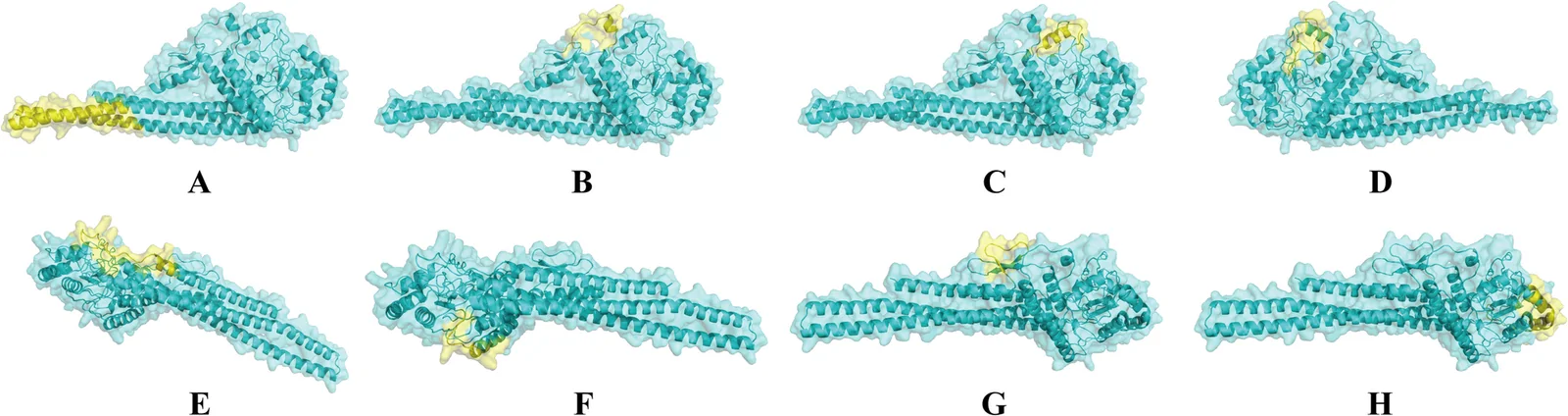
Top eight predicted conformational B-cell epitope clusters mapped onto the refined three-dimensional model of MCV1. Surface-exposed residues belonging to the predicted conformational B-cell epitope clusters are highlighted on the MCV1 structure

A total of 14 conformational B-cell epitopes were identified in MCV1, and 8 in MCV2. The top 8 highest-scoring conformational B-cell epitopes of MCV1 had 12 to 68 amino acid residues, with scores ranging from 0.676 to 0.846 (Fig. 10). The top 8 highest-scoring conformational B-cell epitopes of MCV2 had 15 to 55 amino acid residues, with scores ranging from 0.639 to 0.780 (Fig. S7). Detailed information for all predicted conformational B-cell epitopes is provided in Supplementary Table S5.

### Final mRNA sequence design, codon optimization, and in silico cloning

The final mRNA-oriented fusion proteins were arranged as tPA signal peptide–EAAAK linker–vaccine core–EAAAK linker–HLA-B-derived MHC class I trafficking signal. The final MCV1 and MCV2 fusion proteins contained 618 and 588 amino acids, respectively, and neither construct contained the C-terminal 6×His tag used in the core protein models for structural evaluation. The corresponding GenSmart-optimized CDSs were 1854 bp for MCV1 and 1764 bp for MCV2.

Human codon optimization produced GC contents of 61.06% for MCV1 and 60.94% for MCV2. Independent evaluation using the BiologicsCorp CAI Calculator with *Homo sapiens* (Mammalian/HEK) as the reference set yielded CAI values of 0.92 and 0.91, respectively. Neither optimized CDS contained an internal BamHI or XhoI restriction site. These findings indicate favorable predicted adaptation to the selected human codon-usage reference set but do not directly demonstrate expression efficiency in mammalian cells.

**Fig. 11:**
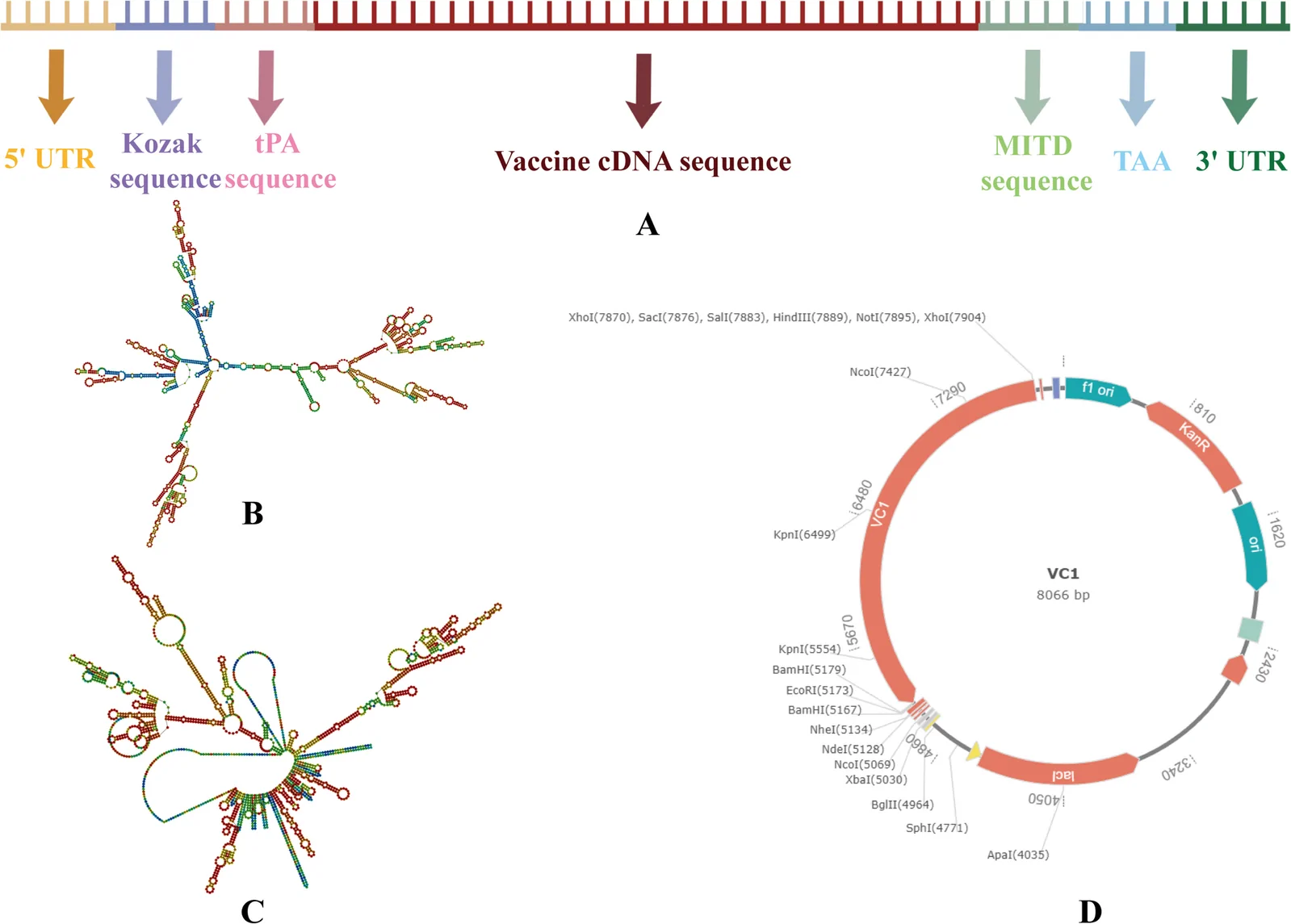
In silico design and sequence-level evaluation of the final MCV1 mRNA construct. **A** Schematic organization of the analyzed MCV1 transcript sequence, comprising the 5′ UTR, Kozak sequence, GenSmart-optimized CDS encoding the tPA signal peptide–EAAAK linker–vaccine core–EAAAK linker–HLA-B-derived MHC class I trafficking signal, TAA stop codon, and 3′ UTR. The 5′ cap and poly(A) tail are not shown; **B** Minimum-free-energy secondary structure predicted by RNAfold (MFE, − 837.70 kcal/mol); **C** Centroid secondary structure predicted by RNAfold (centroid-structure energy, − 610.28 kcal/mol); **D** In silico insertion of the complete MCV1 DNA insert into the pET-28a(+) backbone for IVT-template plasmid construction

RNAfold analysis was performed using the final transcript sequences comprising the 5′ UTR, Kozak sequence, GenSmart-optimized CDS, TAA stop codon, and 3′ UTR, while excluding the 5′ cap and poly(A) tail. The analyzed transcript lengths were 2111 nt for MCV1 and 2021 nt for MCV2. The predicted MFE values were − 837.70 and − 784.90 kcal/mol, respectively, and the corresponding thermodynamic-ensemble free energies were − 863.26 and − 814.59 kcal/mol. The centroid-structure energies were − 610.28 kcal/mol for MCV1 and − 655.40 kcal/mol for MCV2. After normalization for transcript length, the MFE values were − 39.68 and − 38.84 kcal/mol per 100 nt, respectively, indicating broadly comparable predicted folding tendencies under the RNAfold model (Fig. 11A-C; Fig. S8A, B).

Finally, the complete MCV1 and MCV2 IVT DNA inserts were cloned in silico into the pET-28a(+) backbone using flanking BamHI and XhoI sites (Fig. 11D; Fig. S8C). These plasmid constructs were designed for DNA-template propagation and IVT-template preparation, rather than for recombinant antigen expression in *E. coli*.

## Discussion

In this study, we established an immunoinformatics workflow to design and computationally evaluate two bivalent multi-epitope mRNA vaccine constructs targeting *M. pneumoniae* and *C. pneumoniae*. Unlike most previous immunoinformatics-based vaccine studies for these pathogens, which have mainly focused on monovalent, single-pathogen, or single-antigen designs, our study proposes a bivalent multi-epitope mRNA design framework intended to better address clinically relevant coinfection scenarios [9, 10]. Specifically, we selected epitopes from five biologically relevant proteins (P1 and P30 from *M. pneumoniae*; MOMP, OmcA, and OmcB from *C. pneumoniae*) to broaden antigenic coverage and better reflect the clinical context of mixed respiratory infections.

Existing studies have explored single-pathogen vaccines against *M. pneumoniae* or *C. pneumoniae* using immunoinformatics approaches: Pan et al. designed a multi-epitope vaccine targeting the *M. pneumoniae* P30 adhesin with preliminary predicted immunoprotective potential, while Behzad Shahbazi et al. established a similar single-pathogen multi-epitope candidate for *M. pneumoniae* via comprehensive immunoinformatics strategies [10, 67]. For *C. pneumoniae*, Fatima Noor et al. developed a multi-epitope vaccine that induced moderate-intensity immune responses in silico, and Guo et al. constructed a MOMP-targeted multi-epitope candidate that provided a foundational reference for *C. pneumoniae* vaccine development [9, 68].

However, notable gaps remain in these prior works: there are no established bivalent vaccine designs targeting both *M. pneumoniae* and *C. pneumoniae* to address coinfection challenges, the antigenic potential of *C. pneumoniae* proteins such as OmcA and OmcB remains under-explored, and most single-pathogen designs have not systematically addressed potential synergistic immune effects between antigens, adaptation to population-level HLA polymorphism, or the maintenance of long-term immune memory [9, 10]. Compared with these previously reported monovalent immunoinformatics vaccine designs, our study differs mainly in its bivalent antigen composition and mRNA-oriented construct architecture rather than in claiming superior predicted efficacy per se. Similarly, our docking, MD, and immune simulation results were generally within the range commonly reported in published in silico vaccine studies for respiratory pathogens, but such cross-study comparisons should be interpreted cautiously due to substantial differences in underlying modeling pipelines, selected receptors, and simulation settings across publications.

The final epitope panel exhibited favorable predicted profiles: all retained CTL, HTL, and LBL epitopes were antigenic, non-toxic, non-allergenic, and ≥ 90% conserved across the analyzed strain datasets. This stringent filtering pipeline minimized the risk of including unstable or poorly conserved candidates while preserving epitopes driving both cellular and humoral immunity. The core protein constructs also showed favorable predicted antigenicity and contained no abnormal endogenous signal peptides or predicted transmembrane helices before the addition of the exogenous tPA signal peptide and HLA-B-derived trafficking signal. Collectively, these findings indicate consistency with the predefined screening criteria, although they represent computational prioritization rather than direct evidence of biological efficacy in vivo [79].

Structural analysis yielded two key observations. First, refined tertiary structure models of both constructs had acceptable overall quality. While MCV2 had a slightly lower ERRAT score that fell marginally below our preferred screening threshold, its Ramachandran plot was favorable, ProSA-web Z-score remained within the range for native proteins, and low-quality regions were restricted mainly to linker segments rather than the principal functional regions of the constructs. We therefore deemed the MCV2 model acceptable for downstream analysis, while noting it is not equivalent to a high-confidence experimentally resolved structure [80]. Second, both constructs had high theoretical isoelectric points (MCV1: 11.04; MCV2: 10.84), indicating strongly basic proteins. This property may complicate recombinant protein expression, purification, and formulation for optional in vitro validation, as highly basic proteins often show pH-dependent solubility and purification behavior [81]. However, this is of limited relevance to our targeted mRNA platform, where antigens are translated in mammalian cells rather than produced as purified recombinant proteins, though it remains a practical consideration for downstream biochemical characterization.

Exploratory disulfide-bond engineering identified candidate structural variants with improved predicted structural-quality metrics; however, these mutations were evaluated only at the structure-model level and were not incorporated into the final mRNA-encoded sequences. While this design mitigates the risk of local structural changes altering epitope exposure, it cannot fully eliminate this possibility. Similarly, conformational B-cell epitope prediction identified multiple surface-exposed, antibody-accessible regions in both constructs, but these predictions are inherently dependent on the accuracy of our tertiary structure models [82]. Accordingly, our structural analyses only provide supportive evidence for construct plausibility, not definitive validation of native folding or epitope accessibility.

Receptor interaction data require careful interpretation. For MCV1, TLR2 docking is biologically relevant because its β-defensin-3 module is associated with TLR2-mediated innate immune activation [41]. In contrast, MCV2–TLR4 docking was included only as an exploratory comparative analysis and should not be interpreted as evidence of a defined innate activation mechanism for the P2-containing construct. The replicate MD and MM-PBSA analyses enabled assessment of trajectory-level consistency and heterogeneity. MCV1–TLR2 showed relatively limited between-replicate dispersion, whereas the exploratory MCV2–TLR4 system showed numerically greater trajectory-level heterogeneity under the present simulation settings. Because the two complexes involved different receptor systems, these observations were interpreted descriptively and were not used to rank the vaccine constructs. Accordingly, the docking, NMA, replicate MD, and MM-PBSA results support computational interaction plausibility under the specified modeling conditions but do not confirm physiological receptor binding or innate immune activation. These findings remain dependent on modeled structures, receptor templates, docking poses, force-field settings, and finite simulation times and should therefore be regarded as hypothesis-generating rather than mechanistic validation [83].

Immune simulation generated broadly similar modeled response profiles for the two constructs. Descriptive benchmarking showed a numerically higher simulated total-antibody peak for MCV1, broadly similar memory B-cell, helper T-cell, and IFN-γ peaks, and a slightly higher simulated IL-2 peak for MCV2. These differences were not subjected to inferential statistical testing and should not be interpreted as evidence that either construct is immunologically superior. The C-ImmSim platform relies on a simplified mathematical representation of the immune system and cannot fully reproduce tissue-specific antigen expression, innate sensing of mRNA delivery systems, germinal-center dynamics, or host-specific immunoregulatory heterogeneity. Therefore, the modeled antibody, cytokine, and cellular profiles should be regarded as hypothesis-generating rather than predictive of actual in vivo vaccine performance [84]. The benchmarking analysis provides comparative in silico context but does not constitute experimental validation.

Population coverage analysis yielded informative but mixed results. While the selected T-cell epitope panel had high predicted global population coverage, regional analysis revealed marked heterogeneity: coverage was high in North America, Europe, and parts of Africa and South Asia, but reduced in East Asia, South Africa, and Southeast Asia. Notably, the global and regional coverage profiles of our epitope panel are comparable to those reported in recent multi-epitope vaccine studies for respiratory pathogens, with similar patterns of regional heterogeneity observed across published works [66, 85]. This demonstrates that despite favorable global coverage, the epitope panel does not provide equal breadth across all populations, and global coverage metrics alone can mask regional underrepresentation [85]. For future refinement, particularly for region-specific applications, the epitope panel can be optimized to prioritize alleles that improve coverage in underrepresented populations.

mRNA-tailored construct design was a key feature of this study. The final constructs incorporated globin-derived untranslated regions, a Kozak sequence, an N-terminal tPA signal peptide, and a C-terminal HLA-B-derived MHC class I trafficking signal to support the intended translation and intracellular processing of the encoded antigens in mammalian cells [86]. Human codon optimization produced CAI values of 0.92 and 0.91, GC contents of approximately 61%, and broadly comparable length-normalized RNAfold MFE values for MCV1 and MCV2. These findings support the sequence-level plausibility of the constructs but do not directly demonstrate mRNA stability or antigen expression. Codon optimization targeted human expression, whereas pET-28a(+) was used only for the in silico construction of an IVT-template plasmid. Moreover, cap chemistry, poly(A)-tail design, nucleoside modification, and mRNA delivery formulation were not evaluated and remain essential considerations for experimental translation [87].

More broadly, multifunctional studies combining antimicrobial, antiviral, antioxidant, and other biological assays with docking-based analyses illustrate the value of using computational methods to prioritize candidates and support mechanistic interpretation rather than to replace functional testing [88, 89]. Similarly, an integrated study of clove oil and selenium nanoparticles used computational analyses to interpret experimentally observed antifungal activity, further demonstrating that in silico findings are most appropriately framed as mechanistic support rather than direct proof of biological efficacy [90]. A more directly respiratory-pathogen-related example is provided by quercetin complexes against Streptococcus pneumoniae, for which antimicrobial and synergy testing, time-kill analysis, molecular docking, and biofilm-inhibition assays were integrated to connect computational predictions with direct functional evidence [91]. Collectively, these studies reinforce the need for stepwise experimental validation of the present vaccine candidates.

Limitations and future directions: This study is entirely computational and therefore does not establish mRNA expression, antigen presentation, immunogenicity, protective efficacy, or safety. Additional limitations include incomplete representation of pathogen and host diversity, uncertainty associated with predicted protein structures and receptor-interaction models, regional heterogeneity in HLA population coverage, and the absence of an experimentally optimized mRNA delivery system. Several screening decisions were also based on fixed thresholds and single primary prediction platforms, and formal sensitivity analyses across alternative cutoffs or synonymous coding sequences were not performed. Accordingly, the reported CAI, GC-content, RNAfold, docking, MD, MM-PBSA, and immune-simulation results should be regarded as computational design descriptors rather than experimental validation [79, 83].

Therefore, future work should focus on a systematic validation pipeline, including (1) verification of mRNA translation and antigen expression in mammalian cells using Western blotting, immunofluorescence, or flow cytometry after in vitro transfection; (2) assessment of MHC-I and MHC-II antigen presentation efficiency, together with epitope-level validation using synthetic peptide pools, HLA-matched PBMCs, IFN-γ ELISPOT, intracellular cytokine staining, or MHC multimer-based assays; (3) evaluation of antigen-specific CD4⁺ and CD8⁺ T-cell responses, cytokine secretion profiles, and humoral immune responses in vitro; and (4) in vivo immunogenicity, safety, biodistribution, and protective efficacy studies in appropriate animal models using an optimized mRNA delivery formulation. Only through such stepwise experimental validation can the translational potential of these computationally prioritized vaccine candidates be properly assessed.

## Conclusion

In this in silico study, two bivalent multi-epitope mRNA vaccine constructs targeting *Mycoplasma pneumoniae* and *Chlamydia pneumoniae* were computationally prioritized. The constructs showed favorable predicted antigenicity, structural plausibility, receptor-interaction profiles, immune-simulation outputs, population coverage, and human codon-adaptation characteristics. These findings do not establish mRNA expression, immunogenicity, protective efficacy, delivery performance, or clinical safety. Experimental validation should first confirm mRNA integrity, translation, and antigen processing in mammalian cells, followed by HLA-restricted epitope assays and in vivo evaluation of immunogenicity, delivery, biodistribution, safety, and protective efficacy using an optimized mRNA formulation.

## Supplementary Information

Supplementary Material 1.

## Data Availability

All datasets generated or analyzed during the current study are publicly available in Zenodo. The main computational data and analysis results supporting the immunoinformatics-based vaccine design workflow are available at https://zenodo.org/records/20916140. The additional replicate molecular dynamics simulation and downstream analysis results generated during manuscript revision are available at https://zenodo.org/records/20915754.
