# Supplementary material for: In silico design of bivalent multi-epitope mRNA vaccine candidates against Mycoplasma pneumoniae and Chlamydia pneumoniae
Source: BMC Microbiol. 2026 Aug 6;26:949. doi: 10.1186/s12866-026-05473-y (PMC13729508; doi:10.1186/s12866-026-05473-y)
Supplement: Supplementary file 1 [file 12866_2026_5473_MOESM1_ESM.docx]

Supplementary Material

1. **Supplementary Diagram**

**1.1 Supplementary Figure 1**

**Supplementary Figure 1.** Schematic docking models of the 11 CTL epitopes with their corresponding HLA molecules.

**1.2 Supplementary Figure 2**

**Supplementary Figure 2.** Analysis of Interaction Characteristics Between 11 CTL Epitopes and HLA Molecules Using LigPlot+.

**1.3 Supplementary Figure 3**

**Supplementary Figure 3.** Sequence organization and basic structural characterization of the MCV2 core protein construct containing the tetanus toxin-derived P2 helper-epitope module. (A) Sequence organization; (B) SignalP 6.0 prediction profile; (C) predicted secondary-structure composition; (D) DeepTMHMM topology prediction.

**1.4 Supplementary Figure 4**

**Supplementary Figure 4.** LigPlot+ interaction maps of MCV1 and MCV2 with the selected TLR and HLA molecules. (A) MCV1-TLR2; (B) MCV2-TLR4; (C) MCV1-HLA-A*02:01; (D) MCV2-HLA-A*02:01; (E) MCV1-HLA-DRB1*01:01; (F) MCV2-HLA-DRB1*01:01.

**1.5 Supplementary Figure 5**

**Supplementary Figure 5.** Normal-mode analysis of MCV2-TLR4. (A) Deformability; (B) B-factor; (C) Eigenvalues; (D) Variance; (E) Covariance map; (F) Elastic network.

**1.6 Supplementary Figure 6**

**Supplementary Figure 6.** C-ImmSim-predicted post-immunization immune-response profiles for MCV2. (A) Antibody titers; (B) B cell levels; (C) Plasma cell counts; (D) Helper T (Th) cell levels; (E) Subset populations of Th cells; (F) Cytokine secretion levels; (G) Cytotoxic T lymphocyte levels; (H) NK cell counts; (I) Macrophage counts.

**1.7 Supplementary Figure 7**

**Supplementary Figure 7.** Top 8 highest-scoring conformational B-cell epitopes of MCV2.

**1.8 Supplementary Figure 8**

**Supplementary Figure 8.** RNA secondary-structure prediction and in silico cloning of the final MCV2 mRNA construct; (**A**) Minimum-free-energy secondary structure predicted by RNAfold (MFE, −784.90 kcal/mol); (**B**) Centroid secondary structure predicted by RNAfold (centroid-structure energy, −655.40 kcal/mol); (**C**) In silico insertion of the complete MCV2 DNA insert into the pET-28a(+) backbone for IVT-template plasmid construction.

2. **Supplementary Data**

**2.1 Target protein sequences:**

>sp|P11311|ADP1_MYCPN Adhesin P1 OS=Mycoplasma pneumoniae (strain ATCC 29342 / M129 / Subtype 1) OX=272634 GN=mgpA PE=1 SV=2

MHQTKKTALSKSTWILILTATASLATGLTVVGHFTSTTTTLKRQQFSYTRPDEVALRHTNAINPRLTPWTYRNTSFSSLPLTGENPGAWALVRDNSAKGITAGSGSQQTTYDPTRTEAALTASTTFALRRYDLAGRALYDLDFSKLNPQTPTRDQTGQITFNPFGGFGLSGAAPQQWNEVKNKVPVEVAQDPSNPYRFAVLLVPRSVVYYEQLQRGLGLPQQRTESGQNTSTTGAMFGLKVKNAEADTAKSNEKLQGAEATGSSTTSGSGQSTQRGGSSGDTKVKALKIEVKKKSDSEDNGQLQLEKNDLANAPIKRSEESGQSVQLKADDFGTALSSSGSGGNSNPGSPTPWRPWLATEQIHKDLPKWSASILILYDAPYARNRTAIDRVDHLDPKAMTANYPPSWRTPKWNHHGLWDWKARDVLLQTTGFFNPRRHPEWFDGGQTVADNEKTGFDVDNSENTKQGFQKEADSDKSAPIALPFEAYFANIGNLTWFGQALLVFGGNGHVTKSAHTAPLSIGVFRVRYNATGTSATVTGWPYALLFSGMVNKQTDGLKDLPFNNNRWFEYVPRMAVAGAKFVGRELVLAGTITMGDTATVPRLLYDELESNLNLVAQGQGLLREDLQLFTPYGWANRPDLPIGAWSSSSSSSHNAPYYFHNNPDWQDRPIQNVVDAFIKPWEDKNGKDDAKYIYPYRYSGMWAWQVYNWSNKLTDQPLSADFVNENAYQPNSLFAAILNPELLAALPDKVKYGKENEFAANEYERFNQKLTVAPTQGTNWSHFSPTLSRFSTGFNLVGSVLDQVLDYVPWIGNGYRYGNNHRGVDDITAPQTSAGSSSGISTNTSGSRSFLPTFSNIGVGLKANVQATLGGSQTMITGGSPRRTLDQANLQLWTGAGWRNDKASSGQSDENHTKFTSATGMDQQGQSGTSAGNPDSLKQDNISKSGDSLTTQDGNAIDQQEATNYTNLPPNLTPTADWPNALSFTNKNNAQRAQLFLRGLLGSIPVLVNRSGSDSNKFQATDQKWSYTDLHSDQTKLNLPAYGEVNGLLNPALVETYFGNTRAGGSGSNTTSSPGIGFKIPEQNNDSKATLITPGLAWTPQDVGNLVVSGTTVSFQLGGWLVTFTDFVKPRAGYLGLQLTGLDASDATQRALIWAPRPWAAFRGSWVNRLGRVESVWDLKGVWADQAQSDSQGSTTTATRNALPEHPNALAFQVSVVEASAYKPNTSSGQTQSTNSSPYLHLVKPKKVTQSDKLDDDLKNLLDPNQVRTKLRQSFGTDHSTQPQPQSLKTTTPVFGTSSGNLSSVLSGGGAGGGSSGSGQSGVDLSPVEKVSGWLVGQLPSTSDGNTSSTNNLAPNTNTGNDVVGVGRLSESNAAKMNDDVDGIVRTPLAELLDGEGQTADTGPQSVKFKSPDQIDFNRLFTHPVTDLFDPVTMLVYDQYIPLFIDIPASVNPKMVRLKVLSFDTNEQSLGLRLEFFKPDQDTQPNNNVQVNPNNGDFLPLLTASSQGPQTLFSPFNQWPDYVLPLAITVPIVVIVLSVTLGLAIGIPMHKNKQALKAGFALSNQKVDVLTKAVGSVFKEIINRTGISQAPKRLKQTSAAKPGAPRPPVPPKPGAPKPPVQPPKKPA

>WP_053344075.1 adhesin P1 [Mycoplasmoides pneumoniae]

MHQTKKTALSKSTWILILTATASLATGLTVVGHFTSTTTTLKRQQFSYTRPDEVALRHTNAINPRLTPWTYRNTSFSSLPLTGENPGAWALVRDNSAKGITAGSGSQQTTYDPTRTEAALTASTTFALRRYDLAGRALYDLDFSKLNPQTPTRDQTGQITFNPFGGFGLSGAAPQQWNEVKNKVPVEVAQDPSNPYRFAVLLVPRSVVYYEQLQRGLALPNQGSSSGSDSTNQTGAMFGLKVKDATVDSSKQSTESLKGEESSSSSTTSSTSTTQRGGSSNENKVKALQVAVKKKSGSQGNSGDQGTEQVELESNDLANAPIKRGSNNNQQVQLKADDFGTAPSSSGSGTQDGTPTPWTPWLTTEQIHNDPAKFAASILILYDAPYARNRTAIDRVDHLDPKAMTANYPPSWRTPKWNHHGLWDWKARDVLLQTTGFFNPRRHPEWFDGGQTVADNEKTGFDVDNSENTKQGFQKEADSDKSAPIALPFEAYFANIGNLTWFGQALLVFGGNGHVTKSAHTAPLSIGVFRVRYNATGTSATVTGWPYALLFSGMVNKQTDGLKNLPFNNNRWFEYVPRMAVAGAKFVGRELVLAGTITMGDTATVPRLLYDELESNLNLVAQGQGLLREDLQLFTPYGWANRPDLPIGAWSSSSSSHNAPYYFHNNPDWQDRPIQSVVDAFIKPWEDKNGKDDAKYIYPYRYSGMWAWQVYNWSNKLTDQPLSADFVNENAYQPNSLFAAILNPELLAALPDKVKYGKENEFAANEYERFNQKLTVAPTQGTNWSHFSPTLSRFSTGFNLVGSVLDQVLDYVPWIGNGYRYGNNHRGVDDITAPQTSAGSSSGISTNTSGSRSSLPTFSNIGVGLKANVQATLGGSQTMITGGSPRRTLDQANLQLWTGAGWRNDKASSGQSDDHTKFTSATGMGQQEQSGTSAGNPDSLKQDKISKSGDSLTTQDGNAMDQQEATNYTNLPPNLTPTADWPNALSFTNKNNAQRAQLFLRGLLGSIPVLVNKSGQDDNSKFKAEDQKWSYTDLQSDQTKLNLPAYGEVNGLLNPALVETYFGNTRASGSGSNTTSSPGIGFKIPEQSGTNTTSKAVLITPGLAWTPQDVGNLVVSGTSFSFQLGGWLVTFTDFIKPRAGYLGLQLTGLDASDATQRALIWAPRPWAAFRGSWVNRLGRVESVWDLKGVWADQAQSDSQGSTTTATRDALPEHPNALAFQVSVVEASAYKPNTSSGQTQSTNSSPYLHLVKPKKVIQSDKLDDDLKNLLDPNQVRTKLRQSFGTDHSTQPQPQSLKTTTPVFGTSSGNLSSVLSGGGAGGGSSGSGQSGVDLSPVEKVSGWLVGQLPSTSDGNTSSTNNLAPNTNTGNDVVGVGRLSESNAAKMNDDVDGIVRTPLAELLDGEGQTADTGPQSVKFKSPDQIDFNRLFTHPVTDLFDPVTMLVYDQYIPLFIDIPASVNPKMVRLKVLSFDTNEQSLGLRLEFFKPDQDTQPNNNVQVNPNNGDFLPLLTASSQGPQTLFSPFNQWPDYVLPLAITVPIVVIVLSVTLGLAIGIPMHKNKQALKAGFALSNQKVDVLTKAVGSVFKEIINRTGISQAPKRLKQTSAAKPGAPRPPVPPKPGAPKPPVQPPKKPAWYLWNRS

>sp|P75330|P30_MYCPN P30 adhesin OS=Mycoplasma pneumoniae (strain ATCC 29342 / M129 / Subtype 1) OX=272634 GN=p30 PE=4 SV=1

MKLPPRRKLKLFLLAWMLVLFSALIVLATLILVQHNNTELTEVKSELSPLNVVLHAEEDTVQIQGKPITEQAWFIPTVAGCFGFSALAIILGLAIGLPIVKRKEKRLLEEKERQEQLAEQLQRISAQQEEQQALEQQAAAEAHAEAEVEPAPQPVPVPPQPQVQINFGPRTGFPPQPGMAPRPGMPPHPGMAPRPGFPPQPGMAPRPGMPPHPGMAPRPGFPPQPGMAPRPGMPPHPGMAPRPGFPPQPGMAPRPGMQPPRPGMPPQPGFPPKR

>sp|P27455|MOMP_CHLPN Major outer membrane porin OS=Chlamydia pneumoniae OX=83558 GN=ompA PE=2 SV=1

MKKLLKSALLSAAFAGSVGSLQALPVGNPSDPSLLIDGTIWEGAAGDPCDPCATWCDAISLRAGFYGDYVFDRILKVDAPKTFSMGAKPTGSAAANYTTAVDRPNPAYNKHLHDAEWFTNAGFIALNIWDRFDVFCTLGASNGYIRGNSTAFNLVGLFGVKGTTVNANELPNVSLSNGVVELYTDTSFSWSVGARGALWECGCATLGAEFQYAQSKPKVEELNVICNVSQFSVNKPKGYKGVAFPLPTDAGVATATGTKSATINYHEWQVGASLSYRLNSLVPYIGVQWSRATFDADNIRIAQPKLPTAVLNLTAWNPSLLGNATALSTTDSFSDFMQIVSCQINKFKSRKACGVTVGATLVDADKWSLTAEARLINERAAHVSGQFRF

>sp|Q9Z7Z5|OMCA_CHLPN Small cysteine-rich outer membrane protein OmcA OS=Chlamydia pneumoniae OX=83558 GN=omcA PE=2 SV=1

MKKAVLIAAMFCGVVSLSSCCRIVDCCFEDPCAPSSCNPCEVIRKKERSCGGNACGSYVPSCSNPCGSTECNSQSPQVKGCTSPDGRCKQ

>sp|P23700|OMCB_CHLPN Large cysteine-rich periplasmic protein OmcB OS=Chlamydia pneumoniae OX=83558 GN=omcB PE=2 SV=1

MSKLIRRVVTVLALTSMASCFASGGIEAAVAESLITKIVASAETKPAPVPMTAKKVRLVRRNKQPVEQKSRGAFCDKEFYPCEEGRCQPVEAQQESCYGRLYSVKVNDDCNVEICQSVPEYATVGSPYPIEILAIGKKDCVDVVITQQLPCEAEFVSSDPETTPTSDGKLVWKIDRLGAGDKCKITVWVKPLKEGCCFTAATVCACPELRSYTKCGQPAICIKQEGPDCACLRCPVCYKIEVVNTGSAIARNVTVDNPVPDGYSHASGQRVLSFNLGDMRPGDKKVFTVEFCPQRRGQITNVATVTYCGGHKCSANVTTVVNEPCVQVNISGADWSYVCKPVEYSISVSNPGDLVLHDVVIQDTLPSGVTVLEAPGGEICCNKVVWRIKEMCPGETLQFKLVVKAQVPGRFTNQVAVTSESNCGTCTSCAETTTHWKGLAATHMCVLDTNDPICVGENTVYRICVTNRGSAEDTNVSLILKFSKELQPIASSGPTKGTISGNTVVFDALPKLGSKESVEFSVTLKGIAPGDARGEAILSSDTLTSPVSDTENTHVY

2.2 **β-defensin-3**

GIINTLQKYYCRVRGGRCAVLSCLPKEEQIGKCSTRGRKCCRRK

2.3 **Tetanus toxin immunodominant epitope P2 (GenBank: RXM90438.1)**

QYIKANSKFIGITEL

**2.4 Vaccine sequence:**

>MCV1

MGIINTLQKYYCRVRGGRCAVLSCLPKEEQIGKCSTRGRKCCRRKKEAAAKGENTVYRICVTNRGSGPGPGRTGISQAPKRLKQTSGPGPGLTPWTYRNTSFSSLPGPGPGLRQSFGTDHSTQPQPGPGPGSQQTTYDPTRTEAALAAASTAFNLVGLAAASFSWSVGARAAAIALNIWDRFAAAKAVLIAAMFAAARMAVAGAKFAAAKMNDDVDGIAAASIGVFRVRYAAATGWPYALLFAAAKLFLLAWMLAAAVLFSALIVLAAAFLLAWMLVLKKVDADKWSLTAEARLINKKYHEWQVGASLSYRLNSKKAVLIAAMFCGVVSLSSKKRVLSFNLGDMRPGDKKKKSSDTLTSPVSDTENTHKKMWAWQVYNWSNKLTDQKKGGSQTMITGGSPRRTLKKVGVGRLSESNAAKMNDKKITMGDTATVPRLLYDEKKGSPRRTLDQANLQLWTKKPPRPGMPPQPGFPPKRKKPRPGMQPPRPGMPPQPKKFLLAWMLVLFSALIVLKKLILVQHNNTELTEVKSHHHHHH

>MCV2 MQYIKANSKFIGITELEAAAKGENTVYRICVTNRGSGPGPGRTGISQAPKRLKQTSGPGPGLTPWTYRNTSFSSLPGPGPGLRQSFGTDHSTQPQPGPGPGSQQTTYDPTRTEAALAAASTAFNLVGLAAASFSWSVGARAAAIALNIWDRFAAAKAVLIAAMFAAARMAVAGAKFAAAKMNDDVDGIAAASIGVFRVRYAAATGWPYALLFAAAKLFLLAWMLAAAVLFSALIVLAAAFLLAWMLVLKKVDADKWSLTAEARLINKKYHEWQVGASLSYRLNSKKAVLIAAMFCGVVSLSSKKRVLSFNLGDMRPGDKKKKSSDTLTSPVSDTENTHKKMWAWQVYNWSNKLTDQKKGGSQTMITGGSPRRTLKKVGVGRLSESNAAKMNDKKITMGDTATVPRLLYDEKKGSPRRTLDQANLQLWTKKPPRPGMPPQPGFPPKRKKPRPGMQPPRPGMPPQPKKFLLAWMLVLFSALIVLKKLILVQHNNTELTEVKSHHHHHH

2.5 **tPA sequence (UniProt ID: P00750):**

MDAMKRGLCCVLLLCGAVFVSPS

**2.6 MHC class I trafficking signal sequence:**

The MHC class I trafficking signal (MITD), comprising the transmembrane and cytoplasmic domains of the human HLA-B heavy chain, was derived from the human HLA-B reference transcript (NCBI RefSeq: NM_005514.8; protein accession: NP_005505.2). The 55-amino-acid MITD sequence used in the final vaccine constructs was: IVGIVAGLAVLAVVVIGAVVAAVMCRRKSSGGKGGSYSQAACSDSAQGSDVSLTA

(DNA sequence：

ATCGTGGGCATTGTTGCTGGCCTGGCTGTCCTAGCAGTTGTGGTCATCGGAGCTGTGGTCGCTGCTGTGATGTGTAGGAGGAAGAGTTCAGGTGGAAAAGGAGGGAGCTACTCTCAGGCTGCGTGCAGCGACAGTGCCCAGGGCTCTGATGTGTCTCTCACAGCTTGA)

The corresponding native coding sequence, including the terminal stop codon, was 168 nucleotides in length. The native reference DNA sequence shown above includes its original TGA stop codon. In the final GenSmart-optimized vaccine constructs, the HLA-B-derived trafficking-signal coding region was included without this native stop codon, and a separate TAA stop codon was placed immediately upstream of the 3′ UTR.

**2.7 Kozak sequence:**

GCCACC

**2.8 3’UTR:**

TGACGGGTGGCATCCCTGTGACCCCTCCCCAGTGCCTCTCCTGGCCCTGGAAGTTGCCACTCCAGTGCCCACCAGCCTTGTCCTAATAAAATTAAGTTGCATCAAGCT

**2.9 5’UTR:**

GGACAGATCGCCTGGAGACGCCATCCACGCTGTTTTGACCTCCATAGAAGACACCGGGACCGATCCAGCCTCCGCGGCCGGGAACGGTGCATTGGAACGCGGATTCCCCGTGCCAAGAGTGACTCACCGTCCTTGACACG

**2.10 Stepwise finalization and human codon optimization of MCV1 and MCV2**

The final sequence-level design was completed in a stepwise manner. First, the two vaccine proteins were finalized using the architecture tPA signal peptide–EAAAK linker–vaccine core–EAAAK linker–HLA-B-derived MHC class I trafficking signal. The previously included C-terminal 6×His tag was removed because it was not part of the final mRNA-encoded vaccine antigens. The final fusion proteins contained 618 amino acids for MCV1 and 588 amino acids for MCV2.

Second, the finalized fusion-protein sequences were reverse-translated and codon-optimized for Homo sapiens using the GenSmart™ Codon Optimization Tool. During optimization, the encoded amino acid sequences were preserved, internal BamHI (GGATCC) and XhoI (CTCGAG) restriction sites were excluded, and overall and local GC composition was adjusted according to the GenSmart optimization framework.

Third, the codon adaptation indices of the final GenSmart-optimized coding sequences were independently calculated using the BiologicsCorp Codon Adaptation Index Calculator with Homo sapiens (Mammalian/HEK) selected as the reference set. The final CAI values were 0.92 for MCV1 and 0.91 for MCV2. The corresponding GC contents reported by GenSmart were 61.06% and 60.94%, respectively.

Fourth, each final optimized coding sequence was assembled with the selected 5′ untranslated region, Kozak sequence, TAA stop codon, and 3′ untranslated region to generate the final full-length mRNA transcript for RNA secondary-structure prediction. The 5′ cap and poly(A) tail were not represented in the RNAfold input sequences because the cap cannot be encoded as a conventional nucleotide sequence and the final poly(A)-tail length was not predefined.

Finally, BamHI and XhoI restriction sites were added to the 5′ and 3′ ends, respectively, to generate the final DNA templates for in vitro transcription and in silico plasmid construction. No internal BamHI or XhoI sites were present in either final optimized coding sequence.

**2.11 Final GenSmart-optimized coding sequences of MCV1 and MCV2**

The sequences below represent the final human-optimized coding sequences from the tPA initiation codon to the final codon of the HLA-B-derived MHC class I trafficking signal. They do not contain untranslated regions, the Kozak sequence, stop codon, restriction sites, or poly(A) tail.

>MCV1_GenSmart_optimized_CDS_no_stop

ATGGACGCCATGAAGCGTGGCCTGTGCTGCGTGCTGCTGCTGTGCGGTGCCGTGTTCGTGTCTCCCAGCGAGGCCGCCGCCAAGATGGGCATTATCAACACACTGCAGAAATACTACTGCAGAGTGCGGGGCGGGCGCTGCGCTGTGCTTTCTTGTCTGCCTAAGGAAGAGCAGATCGGCAAGTGCAGCACCAGAGGAAGAAAGTGTTGTAGACGCAAGAAAGAAGCCGCTGCCAAGGGCGAGAACACCGTGTACCGGATCTGCGTGACCAACAGAGGCTCTGGCCCTGGCCCTGGACGGACAGGAATCAGCCAAGCCCCTAAGCGGCTGAAGCAGACCAGCGGCCCAGGCCCCGGACTGACACCTTGGACTTATAGAAACACATCATTTAGCAGCCTGCCCGGCCCTGGCCCTGGCCTGAGACAGAGCTTCGGCACCGACCATAGCACACAGCCTCAACCTGGACCCGGCCCAGGTTCCCAGCAGACCACCTACGACCCCACCAGAACCGAGGCCGCCCTGGCCGCCGCCAGCACAGCCTTCAATCTGGTCGGCCTGGCCGCAGCCTCTTTCAGCTGGAGCGTGGGCGCTCGGGCCGCCGCCATCGCCCTGAACATCTGGGACAGATTCGCCGCCGCCAAGGCCGTGTTGATCGCCGCCATGTTCGCCGCTGCCAGGATGGCCGTGGCTGGCGCCAAGTTCGCCGCTGCCAAGATGAACGACGACGTGGACGGCATCGCCGCCGCTAGCATCGGCGTGTTCAGAGTGCGGTACGCCGCCGCAACCGGCTGGCCTTACGCCCTGCTGTTCGCCGCTGCCAAACTGTTTCTGCTGGCTTGGATGCTGGCCGCCGCTGTTCTGTTCAGCGCCCTCATTGTGCTGGCTGCCGCCTTTCTGCTGGCCTGGATGCTGGTGCTGAAAAAGGTGGATGCTGATAAGTGGAGCCTGACAGCAGAAGCTAGACTGATCAACAAGAAATACCACGAGTGGCAGGTGGGCGCCAGCCTGTCTTACAGACTGAATAGCAAAAAGGCCGTCCTCATCGCCGCAATGTTCTGCGGCGTGGTGTCCCTGAGCAGCAAAAAGAGAGTGCTGAGCTTTAATCTGGGCGATATGAGACCTGGTGACAAGAAGAAGAAGAGCAGCGACACCCTGACATCTCCTGTGTCTGATACCGAAAACACCCACAAGAAGATGTGGGCCTGGCAGGTGTACAACTGGTCCAACAAACTGACAGACCAGAAGAAAGGAGGCAGCCAGACCATGATCACCGGCGGCAGCCCTAGAAGAACGCTGAAAAAAGTGGGAGTGGGCAGACTGTCTGAGAGCAACGCCGCCAAGATGAATGATAAGAAGATCACCATGGGCGACACAGCCACCGTGCCTAGACTGCTGTACGACGAGAAGAAGGGTTCCCCTAGACGGACCCTGGACCAGGCCAACCTTCAGCTGTGGACCAAGAAGCCACCTCGGCCCGGCATGCCCCCCCAGCCCGGCTTCCCCCCCAAGCGGAAGAAGCCTAGACCTGGAATGCAGCCTCCACGGCCTGGCATGCCTCCACAGCCTAAGAAGTTCCTGCTCGCCTGGATGCTGGTGCTGTTCAGCGCCCTGATCGTGCTGAAGAAATTGATCCTGGTGCAGCACAACAACACCGAACTGACAGAGGTGAAGTCCGAGGCCGCCGCTAAGATCGTGGGCATCGTTGCCGGCCTGGCTGTGCTGGCCGTCGTGGTCATCGGCGCTGTGGTGGCCGCCGTGATGTGCAGAAGAAAGAGCTCTGGCGGCAAGGGCGGCAGCTACTCCCAAGCCGCTTGTAGCGACAGCGCCCAGGGCAGCGATGTGAGCCTGACCGCC

>MCV2_GenSmart_optimized_CDS_no_stop

ATGGACGCCATGAAGCGGGGCCTGTGCTGTGTGCTGCTGCTGTGTGGCGCCGTGTTCGTGTCCCCATCTGAAGCCGCCGCCAAGATGCAGTACATCAAGGCCAATAGCAAGTTCATCGGCATCACCGAGCTGGAAGCCGCGGCCAAGGGCGAGAATACCGTGTACAGAATCTGCGTGACCAACAGAGGCTCTGGCCCGGGCCCTGGTAGAACAGGCATCAGCCAGGCCCCTAAGCGGCTGAAGCAGACCAGCGGCCCCGGCCCTGGCCTGACACCTTGGACATACAGAAATACCAGCTTCAGCTCTCTGCCTGGCCCTGGCCCCGGCCTGAGACAGAGCTTCGGCACAGACCACAGCACACAGCCCCAGCCTGGCCCCGGCCCAGGGAGCCAGCAGACCACATACGACCCCACCAGAACCGAAGCCGCCCTGGCAGCTGCGAGCACCGCCTTCAACCTGGTGGGCCTGGCCGCTGCCTCTTTCAGCTGGAGCGTCGGAGCTAGAGCCGCCGCCATCGCCCTGAACATCTGGGATCGTTTCGCCGCCGCCAAGGCAGTGCTGATCGCCGCCATGTTCGCCGCCGCTAGAATGGCCGTTGCCGGCGCCAAGTTCGCCGCCGCTAAGATGAACGATGACGTGGACGGAATTGCCGCCGCTTCTATCGGAGTATTTAGAGTGCGGTACGCCGCCGCCACCGGATGGCCCTACGCCCTTCTGTTCGCCGCTGCCAAACTGTTCCTGCTGGCCTGGATGCTGGCCGCCGCAGTGCTGTTCTCCGCCCTGATCGTGCTGGCCGCTGCTTTCCTGCTGGCCTGGATGCTGGTCTTAAAGAAGGTGGACGCCGATAAGTGGTCCCTGACAGCCGAGGCAAGACTGATCAACAAGAAGTACCACGAGTGGCAGGTGGGCGCCAGCCTGAGCTACAGACTGAATAGCAAGAAGGCCGTGCTGATCGCCGCTATGTTCTGCGGCGTGGTCAGCCTGTCTTCTAAAAAGCGGGTGCTGAGCTTTAACCTGGGCGACATGAGGCCCGGCGATAAGAAAAAGAAGTCCAGCGACACCCTGACCTCACCAGTGTCCGATACCGAAAACACCCATAAGAAAATGTGGGCCTGGCAAGTGTACAACTGGAGCAACAAGCTGACAGACCAGAAGAAGGGCGGCAGCCAGACCATGATCACTGGAGGCAGCCCCAGACGGACACTGAAAAAGGTGGGCGTGGGCAGACTGAGCGAGAGCAACGCCGCCAAAATGAACGACAAGAAGATCACCATGGGCGACACCGCCACAGTGCCTAGGCTCCTTTATGACGAGAAGAAGGGAAGCCCTAGACGGACCCTGGACCAAGCCAACCTGCAGCTGTGGACCAAGAAACCTCCTCGGCCTGGCATGCCTCCTCAGCCTGGCTTTCCACCTAAAAGAAAGAAACCTCGGCCCGGCATGCAGCCTCCTAGACCTGGAATGCCCCCTCAACCAAAGAAGTTTCTGCTGGCTTGGATGCTGGTGCTGTTCAGCGCTCTCATCGTGCTGAAGAAGCTGATTCTGGTCCAGCACAACAACACCGAACTGACAGAGGTGAAGTCTGAGGCCGCTGCTAAGATCGTGGGCATCGTTGCTGGCCTGGCGGTGCTAGCCGTGGTGGTGATCGGCGCCGTGGTGGCCGCCGTGATGTGCAGAAGAAAGAGCAGCGGAGGAAAGGGCGGAAGCTACAGCCAGGCTGCATGCAGCGACTCTGCCCAGGGCAGTGATGTGTCCCTGACCGCC

**2.12 Final full-length mRNA transcript sequences used for RNAfold**

The sequences below were used for RNAfold analysis and contain, in order, the 5′ UTR, Kozak sequence, final GenSmart-optimized CDS, TAA stop codon, and 3′ UTR. They are shown as RNA sequences and therefore use U instead of T. The chemically added 5′ cap and poly(A) tail were not included.

>MCV1_full_mRNA_for_RNAfold_no_cap_no_polyA

GGACAGAUCGCCUGGAGACGCCAUCCACGCUGUUUUGACCUCCAUAGAAGACACCGGGACCGAUCCAGCCUCCGCGGCCGGGAACGGUGCAUUGGAACGCGGAUUCCCCGUGCCAAGAGUGACUCACCGUCCUUGACACGGCCACCAUGGACGCCAUGAAGCGUGGCCUGUGCUGCGUGCUGCUGCUGUGCGGUGCCGUGUUCGUGUCUCCCAGCGAGGCCGCCGCCAAGAUGGGCAUUAUCAACACACUGCAGAAAUACUACUGCAGAGUGCGGGGCGGGCGCUGCGCUGUGCUUUCUUGUCUGCCUAAGGAAGAGCAGAUCGGCAAGUGCAGCACCAGAGGAAGAAAGUGUUGUAGACGCAAGAAAGAAGCCGCUGCCAAGGGCGAGAACACCGUGUACCGGAUCUGCGUGACCAACAGAGGCUCUGGCCCUGGCCCUGGACGGACAGGAAUCAGCCAAGCCCCUAAGCGGCUGAAGCAGACCAGCGGCCCAGGCCCCGGACUGACACCUUGGACUUAUAGAAACACAUCAUUUAGCAGCCUGCCCGGCCCUGGCCCUGGCCUGAGACAGAGCUUCGGCACCGACCAUAGCACACAGCCUCAACCUGGACCCGGCCCAGGUUCCCAGCAGACCACCUACGACCCCACCAGAACCGAGGCCGCCCUGGCCGCCGCCAGCACAGCCUUCAAUCUGGUCGGCCUGGCCGCAGCCUCUUUCAGCUGGAGCGUGGGCGCUCGGGCCGCCGCCAUCGCCCUGAACAUCUGGGACAGAUUCGCCGCCGCCAAGGCCGUGUUGAUCGCCGCCAUGUUCGCCGCUGCCAGGAUGGCCGUGGCUGGCGCCAAGUUCGCCGCUGCCAAGAUGAACGACGACGUGGACGGCAUCGCCGCCGCUAGCAUCGGCGUGUUCAGAGUGCGGUACGCCGCCGCAACCGGCUGGCCUUACGCCCUGCUGUUCGCCGCUGCCAAACUGUUUCUGCUGGCUUGGAUGCUGGCCGCCGCUGUUCUGUUCAGCGCCCUCAUUGUGCUGGCUGCCGCCUUUCUGCUGGCCUGGAUGCUGGUGCUGAAAAAGGUGGAUGCUGAUAAGUGGAGCCUGACAGCAGAAGCUAGACUGAUCAACAAGAAAUACCACGAGUGGCAGGUGGGCGCCAGCCUGUCUUACAGACUGAAUAGCAAAAAGGCCGUCCUCAUCGCCGCAAUGUUCUGCGGCGUGGUGUCCCUGAGCAGCAAAAAGAGAGUGCUGAGCUUUAAUCUGGGCGAUAUGAGACCUGGUGACAAGAAGAAGAAGAGCAGCGACACCCUGACAUCUCCUGUGUCUGAUACCGAAAACACCCACAAGAAGAUGUGGGCCUGGCAGGUGUACAACUGGUCCAACAAACUGACAGACCAGAAGAAAGGAGGCAGCCAGACCAUGAUCACCGGCGGCAGCCCUAGAAGAACGCUGAAAAAAGUGGGAGUGGGCAGACUGUCUGAGAGCAACGCCGCCAAGAUGAAUGAUAAGAAGAUCACCAUGGGCGACACAGCCACCGUGCCUAGACUGCUGUACGACGAGAAGAAGGGUUCCCCUAGACGGACCCUGGACCAGGCCAACCUUCAGCUGUGGACCAAGAAGCCACCUCGGCCCGGCAUGCCCCCCCAGCCCGGCUUCCCCCCCAAGCGGAAGAAGCCUAGACCUGGAAUGCAGCCUCCACGGCCUGGCAUGCCUCCACAGCCUAAGAAGUUCCUGCUCGCCUGGAUGCUGGUGCUGUUCAGCGCCCUGAUCGUGCUGAAGAAAUUGAUCCUGGUGCAGCACAACAACACCGAACUGACAGAGGUGAAGUCCGAGGCCGCCGCUAAGAUCGUGGGCAUCGUUGCCGGCCUGGCUGUGCUGGCCGUCGUGGUCAUCGGCGCUGUGGUGGCCGCCGUGAUGUGCAGAAGAAAGAGCUCUGGCGGCAAGGGCGGCAGCUACUCCCAAGCCGCUUGUAGCGACAGCGCCCAGGGCAGCGAUGUGAGCCUGACCGCCUAAUGACGGGUGGCAUCCCUGUGACCCCUCCCCAGUGCCUCUCCUGGCCCUGGAAGUUGCCACUCCAGUGCCCACCAGCCUUGUCCUAAUAAAAUUAAGUUGCAUCAAGCU

>MCV2_full_mRNA_for_RNAfold_no_cap_no_polyA

GGACAGAUCGCCUGGAGACGCCAUCCACGCUGUUUUGACCUCCAUAGAAGACACCGGGACCGAUCCAGCCUCCGCGGCCGGGAACGGUGCAUUGGAACGCGGAUUCCCCGUGCCAAGAGUGACUCACCGUCCUUGACACGGCCACCAUGGACGCCAUGAAGCGGGGCCUGUGCUGUGUGCUGCUGCUGUGUGGCGCCGUGUUCGUGUCCCCAUCUGAAGCCGCCGCCAAGAUGCAGUACAUCAAGGCCAAUAGCAAGUUCAUCGGCAUCACCGAGCUGGAAGCCGCGGCCAAGGGCGAGAAUACCGUGUACAGAAUCUGCGUGACCAACAGAGGCUCUGGCCCGGGCCCUGGUAGAACAGGCAUCAGCCAGGCCCCUAAGCGGCUGAAGCAGACCAGCGGCCCCGGCCCUGGCCUGACACCUUGGACAUACAGAAAUACCAGCUUCAGCUCUCUGCCUGGCCCUGGCCCCGGCCUGAGACAGAGCUUCGGCACAGACCACAGCACACAGCCCCAGCCUGGCCCCGGCCCAGGGAGCCAGCAGACCACAUACGACCCCACCAGAACCGAAGCCGCCCUGGCAGCUGCGAGCACCGCCUUCAACCUGGUGGGCCUGGCCGCUGCCUCUUUCAGCUGGAGCGUCGGAGCUAGAGCCGCCGCCAUCGCCCUGAACAUCUGGGAUCGUUUCGCCGCCGCCAAGGCAGUGCUGAUCGCCGCCAUGUUCGCCGCCGCUAGAAUGGCCGUUGCCGGCGCCAAGUUCGCCGCCGCUAAGAUGAACGAUGACGUGGACGGAAUUGCCGCCGCUUCUAUCGGAGUAUUUAGAGUGCGGUACGCCGCCGCCACCGGAUGGCCCUACGCCCUUCUGUUCGCCGCUGCCAAACUGUUCCUGCUGGCCUGGAUGCUGGCCGCCGCAGUGCUGUUCUCCGCCCUGAUCGUGCUGGCCGCUGCUUUCCUGCUGGCCUGGAUGCUGGUCUUAAAGAAGGUGGACGCCGAUAAGUGGUCCCUGACAGCCGAGGCAAGACUGAUCAACAAGAAGUACCACGAGUGGCAGGUGGGCGCCAGCCUGAGCUACAGACUGAAUAGCAAGAAGGCCGUGCUGAUCGCCGCUAUGUUCUGCGGCGUGGUCAGCCUGUCUUCUAAAAAGCGGGUGCUGAGCUUUAACCUGGGCGACAUGAGGCCCGGCGAUAAGAAAAAGAAGUCCAGCGACACCCUGACCUCACCAGUGUCCGAUACCGAAAACACCCAUAAGAAAAUGUGGGCCUGGCAAGUGUACAACUGGAGCAACAAGCUGACAGACCAGAAGAAGGGCGGCAGCCAGACCAUGAUCACUGGAGGCAGCCCCAGACGGACACUGAAAAAGGUGGGCGUGGGCAGACUGAGCGAGAGCAACGCCGCCAAAAUGAACGACAAGAAGAUCACCAUGGGCGACACCGCCACAGUGCCUAGGCUCCUUUAUGACGAGAAGAAGGGAAGCCCUAGACGGACCCUGGACCAAGCCAACCUGCAGCUGUGGACCAAGAAACCUCCUCGGCCUGGCAUGCCUCCUCAGCCUGGCUUUCCACCUAAAAGAAAGAAACCUCGGCCCGGCAUGCAGCCUCCUAGACCUGGAAUGCCCCCUCAACCAAAGAAGUUUCUGCUGGCUUGGAUGCUGGUGCUGUUCAGCGCUCUCAUCGUGCUGAAGAAGCUGAUUCUGGUCCAGCACAACAACACCGAACUGACAGAGGUGAAGUCUGAGGCCGCUGCUAAGAUCGUGGGCAUCGUUGCUGGCCUGGCGGUGCUAGCCGUGGUGGUGAUCGGCGCCGUGGUGGCCGCCGUGAUGUGCAGAAGAAAGAGCAGCGGAGGAAAGGGCGGAAGCUACAGCCAGGCUGCAUGCAGCGACUCUGCCCAGGGCAGUGAUGUGUCCCUGACCGCCUAAUGACGGGUGGCAUCCCUGUGACCCCUCCCCAGUGCCUCUCCUGGCCCUGGAAGUUGCCACUCCAGUGCCCACCAGCCUUGUCCUAAUAAAAUUAAGUUGCAUCAAGCU

**2.13 Final DNA insert sequences for IVT-template plasmid construction**

The sequences below represent the complete DNA inserts used for IVT-template plasmid construction. Each insert contains BamHI, the 5′ UTR, Kozak sequence, optimized CDS, TAA stop codon, 3′ UTR, and XhoI. The T7 promoter is provided by the pET-28a(+) backbone and is therefore not included in the listed insert sequences.

>MCV1_final_IVT_DNA_template

GGATCCGGACAGATCGCCTGGAGACGCCATCCACGCTGTTTTGACCTCCATAGAAGACACCGGGACCGATCCAGCCTCCGCGGCCGGGAACGGTGCATTGGAACGCGGATTCCCCGTGCCAAGAGTGACTCACCGTCCTTGACACGGCCACCATGGACGCCATGAAGCGTGGCCTGTGCTGCGTGCTGCTGCTGTGCGGTGCCGTGTTCGTGTCTCCCAGCGAGGCCGCCGCCAAGATGGGCATTATCAACACACTGCAGAAATACTACTGCAGAGTGCGGGGCGGGCGCTGCGCTGTGCTTTCTTGTCTGCCTAAGGAAGAGCAGATCGGCAAGTGCAGCACCAGAGGAAGAAAGTGTTGTAGACGCAAGAAAGAAGCCGCTGCCAAGGGCGAGAACACCGTGTACCGGATCTGCGTGACCAACAGAGGCTCTGGCCCTGGCCCTGGACGGACAGGAATCAGCCAAGCCCCTAAGCGGCTGAAGCAGACCAGCGGCCCAGGCCCCGGACTGACACCTTGGACTTATAGAAACACATCATTTAGCAGCCTGCCCGGCCCTGGCCCTGGCCTGAGACAGAGCTTCGGCACCGACCATAGCACACAGCCTCAACCTGGACCCGGCCCAGGTTCCCAGCAGACCACCTACGACCCCACCAGAACCGAGGCCGCCCTGGCCGCCGCCAGCACAGCCTTCAATCTGGTCGGCCTGGCCGCAGCCTCTTTCAGCTGGAGCGTGGGCGCTCGGGCCGCCGCCATCGCCCTGAACATCTGGGACAGATTCGCCGCCGCCAAGGCCGTGTTGATCGCCGCCATGTTCGCCGCTGCCAGGATGGCCGTGGCTGGCGCCAAGTTCGCCGCTGCCAAGATGAACGACGACGTGGACGGCATCGCCGCCGCTAGCATCGGCGTGTTCAGAGTGCGGTACGCCGCCGCAACCGGCTGGCCTTACGCCCTGCTGTTCGCCGCTGCCAAACTGTTTCTGCTGGCTTGGATGCTGGCCGCCGCTGTTCTGTTCAGCGCCCTCATTGTGCTGGCTGCCGCCTTTCTGCTGGCCTGGATGCTGGTGCTGAAAAAGGTGGATGCTGATAAGTGGAGCCTGACAGCAGAAGCTAGACTGATCAACAAGAAATACCACGAGTGGCAGGTGGGCGCCAGCCTGTCTTACAGACTGAATAGCAAAAAGGCCGTCCTCATCGCCGCAATGTTCTGCGGCGTGGTGTCCCTGAGCAGCAAAAAGAGAGTGCTGAGCTTTAATCTGGGCGATATGAGACCTGGTGACAAGAAGAAGAAGAGCAGCGACACCCTGACATCTCCTGTGTCTGATACCGAAAACACCCACAAGAAGATGTGGGCCTGGCAGGTGTACAACTGGTCCAACAAACTGACAGACCAGAAGAAAGGAGGCAGCCAGACCATGATCACCGGCGGCAGCCCTAGAAGAACGCTGAAAAAAGTGGGAGTGGGCAGACTGTCTGAGAGCAACGCCGCCAAGATGAATGATAAGAAGATCACCATGGGCGACACAGCCACCGTGCCTAGACTGCTGTACGACGAGAAGAAGGGTTCCCCTAGACGGACCCTGGACCAGGCCAACCTTCAGCTGTGGACCAAGAAGCCACCTCGGCCCGGCATGCCCCCCCAGCCCGGCTTCCCCCCCAAGCGGAAGAAGCCTAGACCTGGAATGCAGCCTCCACGGCCTGGCATGCCTCCACAGCCTAAGAAGTTCCTGCTCGCCTGGATGCTGGTGCTGTTCAGCGCCCTGATCGTGCTGAAGAAATTGATCCTGGTGCAGCACAACAACACCGAACTGACAGAGGTGAAGTCCGAGGCCGCCGCTAAGATCGTGGGCATCGTTGCCGGCCTGGCTGTGCTGGCCGTCGTGGTCATCGGCGCTGTGGTGGCCGCCGTGATGTGCAGAAGAAAGAGCTCTGGCGGCAAGGGCGGCAGCTACTCCCAAGCCGCTTGTAGCGACAGCGCCCAGGGCAGCGATGTGAGCCTGACCGCCTAATGACGGGTGGCATCCCTGTGACCCCTCCCCAGTGCCTCTCCTGGCCCTGGAAGTTGCCACTCCAGTGCCCACCAGCCTTGTCCTAATAAAATTAAGTTGCATCAAGCTCTCGAG

>MCV2_final_IVT_DNA_template

GGATCCGGACAGATCGCCTGGAGACGCCATCCACGCTGTTTTGACCTCCATAGAAGACACCGGGACCGATCCAGCCTCCGCGGCCGGGAACGGTGCATTGGAACGCGGATTCCCCGTGCCAAGAGTGACTCACCGTCCTTGACACGGCCACCATGGACGCCATGAAGCGGGGCCTGTGCTGTGTGCTGCTGCTGTGTGGCGCCGTGTTCGTGTCCCCATCTGAAGCCGCCGCCAAGATGCAGTACATCAAGGCCAATAGCAAGTTCATCGGCATCACCGAGCTGGAAGCCGCGGCCAAGGGCGAGAATACCGTGTACAGAATCTGCGTGACCAACAGAGGCTCTGGCCCGGGCCCTGGTAGAACAGGCATCAGCCAGGCCCCTAAGCGGCTGAAGCAGACCAGCGGCCCCGGCCCTGGCCTGACACCTTGGACATACAGAAATACCAGCTTCAGCTCTCTGCCTGGCCCTGGCCCCGGCCTGAGACAGAGCTTCGGCACAGACCACAGCACACAGCCCCAGCCTGGCCCCGGCCCAGGGAGCCAGCAGACCACATACGACCCCACCAGAACCGAAGCCGCCCTGGCAGCTGCGAGCACCGCCTTCAACCTGGTGGGCCTGGCCGCTGCCTCTTTCAGCTGGAGCGTCGGAGCTAGAGCCGCCGCCATCGCCCTGAACATCTGGGATCGTTTCGCCGCCGCCAAGGCAGTGCTGATCGCCGCCATGTTCGCCGCCGCTAGAATGGCCGTTGCCGGCGCCAAGTTCGCCGCCGCTAAGATGAACGATGACGTGGACGGAATTGCCGCCGCTTCTATCGGAGTATTTAGAGTGCGGTACGCCGCCGCCACCGGATGGCCCTACGCCCTTCTGTTCGCCGCTGCCAAACTGTTCCTGCTGGCCTGGATGCTGGCCGCCGCAGTGCTGTTCTCCGCCCTGATCGTGCTGGCCGCTGCTTTCCTGCTGGCCTGGATGCTGGTCTTAAAGAAGGTGGACGCCGATAAGTGGTCCCTGACAGCCGAGGCAAGACTGATCAACAAGAAGTACCACGAGTGGCAGGTGGGCGCCAGCCTGAGCTACAGACTGAATAGCAAGAAGGCCGTGCTGATCGCCGCTATGTTCTGCGGCGTGGTCAGCCTGTCTTCTAAAAAGCGGGTGCTGAGCTTTAACCTGGGCGACATGAGGCCCGGCGATAAGAAAAAGAAGTCCAGCGACACCCTGACCTCACCAGTGTCCGATACCGAAAACACCCATAAGAAAATGTGGGCCTGGCAAGTGTACAACTGGAGCAACAAGCTGACAGACCAGAAGAAGGGCGGCAGCCAGACCATGATCACTGGAGGCAGCCCCAGACGGACACTGAAAAAGGTGGGCGTGGGCAGACTGAGCGAGAGCAACGCCGCCAAAATGAACGACAAGAAGATCACCATGGGCGACACCGCCACAGTGCCTAGGCTCCTTTATGACGAGAAGAAGGGAAGCCCTAGACGGACCCTGGACCAAGCCAACCTGCAGCTGTGGACCAAGAAACCTCCTCGGCCTGGCATGCCTCCTCAGCCTGGCTTTCCACCTAAAAGAAAGAAACCTCGGCCCGGCATGCAGCCTCCTAGACCTGGAATGCCCCCTCAACCAAAGAAGTTTCTGCTGGCTTGGATGCTGGTGCTGTTCAGCGCTCTCATCGTGCTGAAGAAGCTGATTCTGGTCCAGCACAACAACACCGAACTGACAGAGGTGAAGTCTGAGGCCGCTGCTAAGATCGTGGGCATCGTTGCTGGCCTGGCGGTGCTAGCCGTGGTGGTGATCGGCGCCGTGGTGGCCGCCGTGATGTGCAGAAGAAAGAGCAGCGGAGGAAAGGGCGGAAGCTACAGCCAGGCTGCATGCAGCGACTCTGCCCAGGGCAGTGATGTGTCCCTGACCGCCTAATGACGGGTGGCATCCCTGTGACCCCTCCCCAGTGCCTCTCCTGGCCCTGGAAGTTGCCACTCCAGTGCCCACCAGCCTTGTCCTAATAAAATTAAGTTGCATCAAGCTCTCGAG

**2.14 Amino acid sequences of the final MCV1 and MCV2 fusion proteins**

The final fusion proteins encoded by the optimized coding sequences were arranged as follows: tPA signal peptide–EAAAK linker–vaccine core–EAAAK linker–HLA-B-derived MHC class I trafficking signal. The C-terminal 6×His tag was excluded.

>MCV1_final_fusion_protein

MDAMKRGLCCVLLLCGAVFVSPSEAAAKMGIINTLQKYYCRVRGGRCAVLSCLPKEEQIGKCSTRGRKCCRRKKEAAAKGENTVYRICVTNRGSGPGPGRTGISQAPKRLKQTSGPGPGLTPWTYRNTSFSSLPGPGPGLRQSFGTDHSTQPQPGPGPGSQQTTYDPTRTEAALAAASTAFNLVGLAAASFSWSVGARAAAIALNIWDRFAAAKAVLIAAMFAAARMAVAGAKFAAAKMNDDVDGIAAASIGVFRVRYAAATGWPYALLFAAAKLFLLAWMLAAAVLFSALIVLAAAFLLAWMLVLKKVDADKWSLTAEARLINKKYHEWQVGASLSYRLNSKKAVLIAAMFCGVVSLSSKKRVLSFNLGDMRPGDKKKKSSDTLTSPVSDTENTHKKMWAWQVYNWSNKLTDQKKGGSQTMITGGSPRRTLKKVGVGRLSESNAAKMNDKKITMGDTATVPRLLYDEKKGSPRRTLDQANLQLWTKKPPRPGMPPQPGFPPKRKKPRPGMQPPRPGMPPQPKKFLLAWMLVLFSALIVLKKLILVQHNNTELTEVKSEAAAKIVGIVAGLAVLAVVVIGAVVAAVMCRRKSSGGKGGSYSQAACSDSAQGSDVSLTA

>MCV2_final_fusion_protein

MDAMKRGLCCVLLLCGAVFVSPSEAAAKMQYIKANSKFIGITELEAAAKGENTVYRICVTNRGSGPGPGRTGISQAPKRLKQTSGPGPGLTPWTYRNTSFSSLPGPGPGLRQSFGTDHSTQPQPGPGPGSQQTTYDPTRTEAALAAASTAFNLVGLAAASFSWSVGARAAAIALNIWDRFAAAKAVLIAAMFAAARMAVAGAKFAAAKMNDDVDGIAAASIGVFRVRYAAATGWPYALLFAAAKLFLLAWMLAAAVLFSALIVLAAAFLLAWMLVLKKVDADKWSLTAEARLINKKYHEWQVGASLSYRLNSKKAVLIAAMFCGVVSLSSKKRVLSFNLGDMRPGDKKKKSSDTLTSPVSDTENTHKKMWAWQVYNWSNKLTDQKKGGSQTMITGGSPRRTLKKVGVGRLSESNAAKMNDKKITMGDTATVPRLLYDEKKGSPRRTLDQANLQLWTKKPPRPGMPPQPGFPPKRKKPRPGMQPPRPGMPPQPKKFLLAWMLVLFSALIVLKKLILVQHNNTELTEVKSEAAAKIVGIVAGLAVLAVVVIGAVVAAVMCRRKSSGGKGGSYSQAACSDSAQGSDVSLTA

**3.	Supplementary Tables**

|  | **3.1 Supplementary Table S1.** HLA class I (CTL epitope prediction) and class II (HTL epitope prediction) reference panels. | | | | |
| --- | --- | --- | --- | --- | --- |
| HLA allele | | HLA class | IEDB panel | Peptide length used in this study | Prediction algorithm |
| HLA-A*01:01 | | I | Human 27-allele reference panel | 9 aa | NetMHCpan 4.1 EL |
| HLA-A*02:01 | | I | Human 27-allele reference panel | 9 aa | NetMHCpan 4.1 EL |
| HLA-A*02:03 | | I | Human 27-allele reference panel | 9 aa | NetMHCpan 4.1 EL |
| HLA-A*02:06 | | I | Human 27-allele reference panel | 9 aa | NetMHCpan 4.1 EL |
| HLA-A*03:01 | | I | Human 27-allele reference panel | 9 aa | NetMHCpan 4.1 EL |
| HLA-A*11:01 | | I | Human 27-allele reference panel | 9 aa | NetMHCpan 4.1 EL |
| HLA-A*23:01 | | I | Human 27-allele reference panel | 9 aa | NetMHCpan 4.1 EL |
| HLA-A*24:02 | | I | Human 27-allele reference panel | 9 aa | NetMHCpan 4.1 EL |
| HLA-A*26:01 | | I | Human 27-allele reference panel | 9 aa | NetMHCpan 4.1 EL |
| HLA-A*30:01 | | I | Human 27-allele reference panel | 9 aa | NetMHCpan 4.1 EL |
| HLA-A*30:02 | | I | Human 27-allele reference panel | 9 aa | NetMHCpan 4.1 EL |
| HLA-A*31:01 | | I | Human 27-allele reference panel | 9 aa | NetMHCpan 4.1 EL |
| HLA-A*32:01 | | I | Human 27-allele reference panel | 9 aa | NetMHCpan 4.1 EL |
| HLA-A*33:01 | | I | Human 27-allele reference panel | 9 aa | NetMHCpan 4.1 EL |
| HLA-A*68:01 | | I | Human 27-allele reference panel | 9 aa | NetMHCpan 4.1 EL |
| HLA-A*68:02 | | I | Human 27-allele reference panel | 9 aa | NetMHCpan 4.1 EL |
| HLA-B*07:02 | | I | Human 27-allele reference panel | 9 aa | NetMHCpan 4.1 EL |
| HLA-B*08:01 | | I | Human 27-allele reference panel | 9 aa | NetMHCpan 4.1 EL |
| HLA-B*15:01 | | I | Human 27-allele reference panel | 9 aa | NetMHCpan 4.1 EL |
| HLA-B*35:01 | | I | Human 27-allele reference panel | 9 aa | NetMHCpan 4.1 EL |
| HLA-B*40:01 | | I | Human 27-allele reference panel | 9 aa | NetMHCpan 4.1 EL |
| HLA-B*44:02 | | I | Human 27-allele reference panel | 9 aa | NetMHCpan 4.1 EL |
| HLA-B*44:03 | | I | Human 27-allele reference panel | 9 aa | NetMHCpan 4.1 EL |
| HLA-B*51:01 | | I | Human 27-allele reference panel | 9 aa | NetMHCpan 4.1 EL |
| HLA-B*53:01 | | I | Human 27-allele reference panel | 9 aa | NetMHCpan 4.1 EL |
| HLA-B*57:01 | | I | Human 27-allele reference panel | 9 aa | NetMHCpan 4.1 EL |
| HLA-B*58:01 | | I | Human 27-allele reference panel | 9 aa | NetMHCpan 4.1 EL |
| HLA-DRB1*01:01 | | II | Human 27-allele reference panel | 15 aa | NetMHCIIpan 4.1 EL |
| HLA-DRB1*03:01 | | II | Human 27-allele reference panel | 15 aa | NetMHCIIpan 4.1 EL |
| HLA-DRB1*04:01 | | II | Human 27-allele reference panel | 15 aa | NetMHCIIpan 4.1 EL |
| HLA-DRB1*04:05 | | II | Human 27-allele reference panel | 15 aa | NetMHCIIpan 4.1 EL |
| HLA-DRB1*07:01 | | II | Human 27-allele reference panel | 15 aa | NetMHCIIpan 4.1 EL |
| HLA-DRB1*08:02 | | II | Human 27-allele reference panel | 15 aa | NetMHCIIpan 4.1 EL |
| HLA-DRB1*09:01 | | II | Human 27-allele reference panel | 15 aa | NetMHCIIpan 4.1 EL |
| HLA-DRB1*11:01 | | II | Human 27-allele reference panel | 15 aa | NetMHCIIpan 4.1 EL |
| HLA-DRB1*12:01 | | II | Human 27-allele reference panel | 15 aa | NetMHCIIpan 4.1 EL |
| HLA-DRB1*13:02 | | II | Human 27-allele reference panel | 15 aa | NetMHCIIpan 4.1 EL |
| HLA-DRB1*15:01 | | II | Human 27-allele reference panel | 15 aa | NetMHCIIpan 4.1 EL |
| HLA-DRB3*01:01 | | II | Human 27-allele reference panel | 15 aa | NetMHCIIpan 4.1 EL |
| HLA-DRB3*02:02 | | II | Human 27-allele reference panel | 15 aa | NetMHCIIpan 4.1 EL |
| HLA-DRB4*01:01 | | II | Human 27-allele reference panel | 15 aa | NetMHCIIpan 4.1 EL |
| HLA-DRB5*01:01 | | II | Human 27-allele reference panel | 15 aa | NetMHCIIpan 4.1 EL |
| HLA-DQA1*05:01/DQB1*02:01 | | II | Human 27-allele reference panel | 15 aa | NetMHCIIpan 4.1 EL |
| HLA-DQA1*05:01/DQB1*03:01 | | II | Human 27-allele reference panel | 15 aa | NetMHCIIpan 4.1 EL |
| HLA-DQA1*03:01/DQB1*03:02 | | II | Human 27-allele reference panel | 15 aa | NetMHCIIpan 4.1 EL |
| HLA-DQA1*04:01/DQB1*04:02 | | II | Human 27-allele reference panel | 15 aa | NetMHCIIpan 4.1 EL |
| HLA-DQA1*01:01/DQB1*05:01 | | II | Human 27-allele reference panel | 15 aa | NetMHCIIpan 4.1 EL |
| HLA-DQA1*01:02/DQB1*06:02 | | II | Human 27-allele reference panel | 15 aa | NetMHCIIpan 4.1 EL |
| HLA-DPA1*02:01/DPB1*01:01 | | II | Human 27-allele reference panel | 15 aa | NetMHCIIpan 4.1 EL |
| HLA-DPA1*01:03/DPB1*02:01 | | II | Human 27-allele reference panel | 15 aa | NetMHCIIpan 4.1 EL |
| HLA-DPA1*01:03/DPB1*04:01 | | II | Human 27-allele reference panel | 15 aa | NetMHCIIpan 4.1 EL |
| HLA-DPA1*03:01/DPB1*04:02 | | II | Human 27-allele reference panel | 15 aa | NetMHCIIpan 4.1 EL |
| HLA-DPA1*02:01/DPB1*05:01 | | II | Human 27-allele reference panel | 15 aa | NetMHCIIpan 4.1 EL |
| HLA-DPA1*02:01/DPB1*14:01 | | II | Human 27-allele reference panel | 15 aa | NetMHCIIpan 4.1 EL |

| **3.2 Supplementary Table S2**. ABCpred-derived linear B-cell epitopes and their supportive validation by BepiPred-3.0. | | | | | | | |
| --- | --- | --- | --- | --- | --- | --- | --- |
| Protein | | Rank | Sequence | Start | Score | BepiPred validation | Overlap with BepiPred-3.0 top-50% residue set |
| MOMP | | 9 | VDADKWSLTAEARLIN | 418 | 0.83 | Full support | 16 |
|  | | 10 | YHEWQVGASLSYRLNS | 321 | 0.82 | Full support | 16 |
| OmcA | | 7 | AVLIAAMFCGVVSLSS | 79 | 0.82 | Full support | 16 |
| OmcB | | 9 | RVLSFNLGDMRPGDKK | 343 | 0.85 | Full support | 15 |
|  | | 28 | SSDTLTSPVSDTENTH | 612 | 0.64 | Full support | 16 |
| P1(FH) | | 1 | MWAWQVYNWSNKLTDQ | 735 | 0.95 | Full support | 16 |
| P1(M129) | | 6 | GGSQTMITGGSPRRTL | 934 | 0.9 | Full support | 14 |
|  | | 11 | VGVGRLSESNAAKMND | 1428 | 0.85 | Full support | 16 |
|  | | 12 | ITMGDTATVPRLLYDE | 656 | 0.84 | Full support | 16 |
|  | | 13 | GSPRRTLDQANLQLWT | 943 | 0.83 | Full support | 15 |
| P30 | | 3 | PPRPGMPPQPGFPPKR | 318 | 0.9 | Full support | 16 |
|  | | 4 | PRPGMQPPRPGMPPQP | 312 | 0.89 | Full support | 16 |
|  | | 16 | FLLAWMLVLFSALIVL | 71 | 0.68 | Full support | 16 |
|  |  | 17 | LILVQHNNTELTEVKS | 89 | 0.62 | Full support | 16 |
|  | **Table legend:** Full support indicates that ≥75% of the residues in the epitope fall within the BepiPred-3.0 top-50% residue set. Partial support indicates 30–74% overlap, whereas no clear support indicates <30% overlap. BepiPred-3.0 was used as a supportive validation tool for retained ABCpred-derived epitopes rather than for de novo epitope re-selection. | | | | | | |

| **3.3 Supplementary Table S3**. Deterministic structural-quality metrics of MCV1 and MCV2 models generated by three prediction servers before and after refinement. | | | | | |  |
| --- | --- | --- | --- | --- | --- | --- |
|  | Vaccine | Server | ERRAT | Verify3D | ProSA | |
| Before optimization | MCV1 | AlphaFold 3 | 86.0759 | 12.50% | -3.16 | |
|  |  | Chai-1 | 78.0303 | 58.96% | -7.35 | |
|  |  | Protenix | 59.2803 | 66.42% | -7.57 | |
|  | MCV2 | AlphaFold 3 | 74.8062 | 14.03% | -2.37 | |
|  |  | Chai-1 | 71.2831 | 56.13% | -6.83 | |
|  |  | Protenix | 39.759 | 68.58% | -6.96 | |
| After optimization | MCV1 | AlphaFold 3 | 96.6543 | 20.34% | -2.58 | |
|  |  | Chai-1 | 89.5238 | 58.40% | -7.43 | |
|  |  | Protenix | 79.0076 | 70.34% | -7.63 | |
|  | MCV2 | AlphaFold 3 | 89.4928 | 20.55% | -3.39 | |
|  |  | Chai-1 | 84.375 | 56.13% | -6.84 | |
|  |  | Protenix | 67.6707 | 69.57% | -7.03 | |
| **Table legend:** Values are deterministic outputs generated by the specified structural-assessment tools and do not represent replicate-based or inferential statistical estimates. | | | | | | |

| **3.4 Supplementary Table S4.** Potential disulfide bond sites and characteristic parameters of MCV1 and MCV2 vaccines. | | | | | | | |
| --- | --- | --- | --- | --- | --- | --- | --- |
| Vaccine | Res1 Seq | Res1 AA | Res2 Seq | Res2 AA | Chi3 | Energy | Sum B-Factors |
| MCV1 | 12 | CYS | 41 | CYS | 103.84 | 1.19 | 0 |
|  | 60 | CYS | 325 | CYS | 86.03 | 1.29 | 0 |
|  | 69 | GLY | 131 | GLY | -82.1 | 1.27 | 0 |
|  | 121 | SER | 396 | THR | 94.95 | 1.35 | 0 |
|  | 136 | THR | 392 | GLN | 114.5 | 1.89 | 0 |
|  | 152 | ALA | 275 | MET | -97.48 | 1.73 | 0 |
|  | 245 | ALA | 526 | THR | -94.73 | 1.46 | 0 |
|  | 313 | ASN | 385 | ASP | -100.62 | 1.86 | 0 |
| MCV2 | 18 | ALA | 82 | LEU | 81.51 | 1.22 | 0 |
|  | 143 | ALA | 155 | ALA | 114.51 | 1.58 | 0 |
|  | 149 | TRP | 302 | SER | 84.83 | 1.66 | 0 |
|  | 176 | PHE | 191 | ALA | -90.99 | 1.3 | 0 |
|  | 187 | GLY | 401 | ALA | -102.99 | 1.68 | 0 |
| **Table legend:** Values are deterministic algorithmic or structure-model outputs and do not represent replicate-based statistical estimates. | | | | | | | |

| **3.5 Supplementary Table S5.** Prediction and characteristics of top 8 conformational B-cell epitopes for MCV1/MCV2 vaccines. | | | | | |
| --- | --- | --- | --- | --- | --- |
| Vaccine | Start | End | Peptide | Number of residues | Score |
| MCV1 | 181 | 248 | RFAAAKAVLIAAMFAAARMAVAGAK  FAAAKMNDDVDGIAAASIGVFRVRYA  AATGWPYALLFAAAKLF | 68 | 0.846 |
|  | 343 | 358 | DMRPGDKKKKSSDTLT | 16 | 0.805 |
|  | 75 | 92 | ISQAPKRLKQTSGPGPGL | 18 | 0.767 |
|  | 456 | 470 | LWTKKPPRPGMPPQP | 15 | 0.708 |
|  | 474 | 497 | PKRKKPRPGMQPPRPGMPPQPKKF | 24 | 0.703 |
|  | 377 | 390 | YNWSNKLTDQKKGG | 14 | 0.693 |
|  | 17 | 28 | GRCAVLSCLPKE | 12 | 0.676 |
|  | 398 | 431 | GSPRRTLKKVGVGRLSESNAAKMNDKK  ITMGDTA | 34 | 0.661 |
| MCV2 | 92 | 108 | TQPQPGPGPGSQQTTYD | 17 | 0.78 |
|  | 232 | 286 | ALIVLAAAFLLAWMLVLKKVDADKWSLT  AEARLINKKYHEWQVGASLSYRLNSKK | 55 | 0.767 |
|  | 73 | 87 | SSLPGPGPGLRQSFG | 15 | 0.765 |
|  | 1 | 44 | MQYIKANSKFIGITELEAAAKGENTVYRIC  VTNRGSGPGPGRTG | 44 | 0.739 |
|  | 412 | 456 | KGSPRRTLDQANLQLWTKKPPRPGMPPQP  GFPPKRKKPRPGMQPP | 45 | 0.712 |
|  | 50 | 65 | KRLKQTSGPGPGLTPW | 16 | 0.686 |
|  | 311 | 327 | LGDMRPGDKKKKSSDTL | 17 | 0.651 |
|  | 373 | 390 | TLKKVGVGRLSESNAAKM | 18 | 0.639 |
| **Table legend:** Values are deterministic algorithmic or structure-model outputs and do not represent replicate-based statistical estimates. | | | | | |

|  | **3.6 Supplementary Table S6.** Cluster-level HADDOCK docking statistics of vaccine–receptor complexes. | | | | | | | | | | |
| --- | --- | --- | --- | --- | --- | --- | --- | --- | --- | --- | --- |
|  | Complex | Selected cluster | Cluster size | HADDOCK score | RMSD from overall lowest-energy structure | Van der Waals energy | Electrostatic energy | Desolvation energy | Restraints violation energy | Buried surface area | Z-score |
|  | MCV1–TLR2 | 1 | 20 | -394.3 ± 7.3 | 0.7 ± 0.4 | -166.5 ± 4.4 | -904.5 ± 17.9 | -46.9 ± 3.7 | 0.0 ± 0.0 | 5440.6 ± 88.5 | 0 |
|  | MCV2–TLR4 | 1 | 20 | -399.0 ± 5.5 | 0.3 ± 0.2 | -190.0 ± 5.7 | -632.9 ± 5.3 | -82.5 ± 1.1 | 0.0 ± 0.0 | 5310.3 ± 96.4 | 0 |
|  | MCV1–HLA-A*02:01 | 1 | 20 | -282.3 ± 1.4 | 0.2 ± 0.1 | -125.0 ± 5.1 | -523.6 ± 31.2 | -52.6 ± 3.0 | 0.0 ± 0.0 | 3521.4 ± 62.6 | 0 |
|  | MCV2–HLA-A*02:01 | 1 | 20 | -162.3 ± 1.5 | 0.2 ± 0.1 | -80.5 ± 0.8 | -6.1 ± 2.2 | -80.6 ± 1.7 | 0.0 ± 0.0 | 2095.5 ± 19.9 | 0 |
|  | MCV1–HLA-DRB1*01:01 | 1 | 20 | -655.9 ± 1.1 | 0.4 ± 0.3 | -339.1 ± 1.5 | -775.8 ± 8.4 | -161.6 ± 2.5 | 0.0 ± 0.0 | 8968.9 ± 25.0 | 0 |
|  | MCV2–HLA-DRB1*01:01 | 1 | 20 | -669.0 ± 4.4 | 0.2 ± 0.1 | -355.0 ± 3.1 | -720.8 ± 12.6 | -169.8 ± 2.1 | 0.0 ± 0.0 | 9012.1 ± 49.5 | 0 |
|  | **Table legend:** Mean ± SD values summarize models within the selected HADDOCK cluster and should not be interpreted as biological-replicate statistics. | | | | | | | | | | |

|  | **3.7 Supplementary Table S7.** Final 20 ns trajectory-level statistics of replicate molecular dynamics simulations. | | | | | | | |
| --- | --- | --- | --- | --- | --- | --- | --- | --- |
|  | Complex | Replicate | Window | RMSD, mean ± SD (nm) | RMSD slope (nm/ns) | Rg, mean ± SD (nm) | H-bonds, mean ± SD | Interpretation |
|  | MCV1–TLR2 | Rep 1 | 80–100 ns | 0.739 ± 0.040 | 0.00412 | 4.120 ± 0.020 | 5.25 ± 1.25 | Post-equilibration window used for convergence assessment |
|  | MCV1–TLR2 | Rep 2 | 80–100 ns | 0.603 ± 0.047 | 0.00009 | 4.068 ± 0.028 | 3.55 ± 1.52 | Post-equilibration window used for convergence assessment |
|  | MCV1–TLR2 | Rep 3 | 80–100 ns | 0.876 ± 0.041 | -0.00089 | 4.059 ± 0.028 | 3.42 ± 0.80 | Post-equilibration window used for convergence assessment |
|  | MCV1–TLR2 | Across replicates | 80–100 ns | 0.739 ± 0.136 | 0.00111 | 4.082 ± 0.033 | 4.07 ± 1.02 | Relatively limited between-replicate dispersion and an average late-window RMSD slope close to zero |
|  | MCV2–TLR4 | Rep 1 | 80–100 ns | 1.499 ± 0.070 | 0.00116 | 3.934 ± 0.043 | 8.69 ± 1.77 | Post-equilibration window used for convergence assessment |
|  | MCV2–TLR4 | Rep 2 | 80–100 ns | 0.960 ± 0.071 | -0.00202 | 4.265 ± 0.038 | 19.08 ± 2.04 | Post-equilibration window used for convergence assessment |
|  | MCV2–TLR4 | Rep 3 | 80–100 ns | 1.621 ± 0.037 | 0.00398 | 3.717 ± 0.030 | 17.96 ± 2.03 | Post-equilibration window used for convergence assessment |
|  | MCV2–TLR4 | Across replicates | 80–100 ns | 1.360 ± 0.352 | 0.00104 | 3.972 ± 0.276 | 15.25 ± 5.70 | Numerically larger between-replicate dispersion than MCV1–TLR2, with an average late-window RMSD slope close to zero |
|  | **Table legend:** Values for individual replicates represent mean ± within-trajectory SD during the 80–100-ns window. Across-replicate values represent the mean ± between-replicate SD of the three trajectory-level summaries. Comparisons between MCV1–TLR2 and MCV2–TLR4 are descriptive because the complexes contain different receptor systems; no formal hypothesis testing was performed. | | | | | | | |

|  | **3.8 Supplementary Table S8.** Chain-level replicate-based RMSF summary. | | | | | | | |  |
| --- | --- | --- | --- | --- | --- | --- | --- | --- | --- |
|  | Complex | Chain | n_residues | Mean RMSF across residues (nm) | Median RMSF (nm) | Maximum mean RMSF (nm) | SD of residue-wise mean RMSF (nm) | Interpretation | |
|  | MCV1–TLR2 | MCV1 | 536 | 0.262 | 0.239 | 0.703 | 0.112 | Localized flexible regions in construct | |
|  | MCV1–TLR2 | TLR2 | 546 | 0.245 | 0.227 | 0.591 | 0.084 | Mostly low-to-moderate receptor-chain fluctuation | |
|  | MCV2–TLR4 | MCV2 | 506 | 0.649 | 0.412 | 1.887 | 0.449 | Numerically higher local flexibility in selected MCV2 regions under the present simulation settings | |
|  | MCV2–TLR4 | TLR4_chain1 | 601 | 0.398 | 0.328 | 1.109 | 0.205 | Numerically higher local fluctuation in selected TLR4 chain 1 regions | |
|  | MCV2–TLR4 | TLR4_chain2 | 140 | 0.201 | 0.198 | 0.384 | 0.049 | Lower overall fluctuation | |
|  | **Table legend:** RMSF profiles were calculated separately for each independent trajectory and summarized at the residue and chain levels. Values are descriptive replicate-based summaries. Because the complexes contain different receptor proteins and chain compositions, cross-complex differences were not subjected to inferential hypothesis testing. | | | | | | | | |

| **3.9 Supplementary Table S9.** Replicate-level MM-PBSA binding free-energy components. | | | | | | |
| --- | --- | --- | --- | --- | --- | --- |
| Complex | Replicate | Frames | Component | Average | SD | SEM |
| MCV1–TLR2  MCV1–TLR2  MCV1–TLR2  MCV1–TLR2  MCV1–TLR2  MCV1–TLR2  MCV1–TLR2  MCV1–TLR2  MCV1–TLR2  MCV1–TLR2  MCV1–TLR2  MCV1–TLR2  MCV1–TLR2  MCV1–TLR2  MCV1–TLR2  MCV1–TLR2  MCV1–TLR2  MCV1–TLR2  MCV1–TLR2  MCV1–TLR2  MCV1–TLR2 | Rep 1 | 201 | ΔVDWAALS | -161.92 | 10.4 | 0.73 |
|  | Rep 1 | 201 | ΔEEL | -1929.75 | 74.33 | 5.24 |
|  | Rep 1 | 201 | ΔEPB | 1964.72 | 71.61 | 5.05 |
|  | Rep 1 | 201 | ΔENPOLAR | -18.85 | 0.81 | 0.06 |
|  | Rep 1 | 201 | ΔGGAS | -2091.68 | 73.8 | 5.21 |
|  | Rep 1 | 201 | ΔGSOLV | 1945.87 | 71.49 | 5.04 |
|  | Rep 1 | 201 | ΔTOTAL | -145.81 | 16.9 | 1.19 |
|  | Rep 2 | 201 | ΔVDWAALS | -160.44 | 8.24 | 0.58 |
|  | Rep 2 | 201 | ΔEEL | -1533.7 | 60.41 | 4.26 |
|  | Rep 2 | 201 | ΔEPB | 1589.69 | 57.22 | 4.04 |
|  | Rep 2 | 201 | ΔENPOLAR | -19.72 | 0.64 | 0.05 |
|  | Rep 2 | 201 | ΔGGAS | -1694.14 | 59.81 | 4.22 |
|  | Rep 2 | 201 | ΔGSOLV | 1569.97 | 57.22 | 4.04 |
|  | Rep 2 | 201 | ΔTOTAL | -124.16 | 11.26 | 0.79 |
|  | Rep 3 | 201 | ΔVDWAALS | -98.8 | 7.48 | 0.53 |
|  | Rep 3 | 201 | ΔEEL | -1845.59 | 82.19 | 5.8 |
|  | Rep 3 | 201 | ΔEPB | 1851.59 | 79.46 | 5.6 |
|  | Rep 3 | 201 | ΔENPOLAR | -12.8 | 0.58 | 0.04 |
|  | Rep 3 | 201 | ΔGGAS | -1944.39 | 82.94 | 5.85 |
|  | Rep 3 | 201 | ΔGSOLV | 1838.8 | 79.22 | 5.59 |
|  | Rep 3 | 201 | ΔTOTAL | -105.59 | 11.16 | 0.79 |
| MCV2–TLR4 | Rep 1 | 201 | ΔVDWAALS | -165.95 | 8.86 | 0.62 |
| MCV2–TLR4 | Rep 1 | 201 | ΔEEL | -3497.92 | 114.1 | 8.05 |
| MCV2–TLR4 | Rep 1 | 201 | ΔEPB | 3541.74 | 114.97 | 8.11 |
| MCV2–TLR4 | Rep 1 | 201 | ΔENPOLAR | -19.02 | 0.87 | 0.06 |
| MCV2–TLR4 | Rep 1 | 201 | ΔGGAS | -3663.86 | 116.57 | 8.22 |
| MCV2–TLR4 | Rep 1 | 201 | ΔGSOLV | 3522.72 | 114.65 | 8.09 |
| MCV2–TLR4 | Rep 1 | 201 | ΔTOTAL | -141.14 | 9.74 | 0.69 |
| MCV2–TLR4 | Rep 2 | 201 | ΔVDWAALS | -150.73 | 8.64 | 0.61 |
| MCV2–TLR4 | Rep 2 | 201 | ΔEEL | 320.81 | 58.64 | 4.14 |
| MCV2–TLR4 | Rep 2 | 201 | ΔEPB | -275.14 | 56.74 | 4 |
| MCV2–TLR4 | Rep 2 | 201 | ΔENPOLAR | -17.74 | 0.65 | 0.05 |
| MCV2–TLR4 | Rep 2 | 201 | ΔGGAS | 170.08 | 57.69 | 4.07 |
| MCV2–TLR4 | Rep 2 | 201 | ΔGSOLV | -292.88 | 56.83 | 4.01 |
| MCV2–TLR4 | Rep 2 | 201 | ΔTOTAL | -122.8 | 13.57 | 0.96 |
| MCV2–TLR4 | Rep 3 | 201 | ΔVDWAALS | -137.44 | 8.88 | 0.63 |
| MCV2–TLR4 | Rep 3 | 201 | ΔEEL | 438.69 | 66.47 | 4.69 |
| MCV2–TLR4 | Rep 3 | 201 | ΔEPB | -401.61 | 62.81 | 4.43 |
| MCV2–TLR4 | Rep 3 | 201 | ΔENPOLAR | -16.31 | 0.65 | 0.05 |
| MCV2–TLR4 | Rep 3 | 201 | ΔGGAS | 301.25 | 63.92 | 4.51 |
| MCV2–TLR4 | Rep 3 | 201 | ΔGSOLV | -417.92 | 63.01 | 4.44 |
| MCV2–TLR4 | Rep 3 | 201 | ΔTOTAL | -116.67 | 11.09 | 0.78 |
| **Table legend:** Energy values are reported in kcal·mol⁻¹. Average, SD, and SEM are frame-level summaries calculated from 201 frames within each trajectory. Frames from the same trajectory were not treated as independent replicates; trajectory-level averages were used to calculate the across-replicate summaries reported in **Table 4**. | | | | | | |

| **3.10 Supplementary Table S10.** Descriptive benchmarking of C-ImmSim-predicted immune-response outputs for MCV1, MCV2, and published multi-epitope vaccine constructs. | | | | | | | | |
| --- | --- | --- | --- | --- | --- | --- | --- | --- |
|  | Construct | Source/reference | Antibody peak | Memory B-cell peak | TH-cell peak | IFN-γ peak | IL-2 peak | Overall interpretation |
|  | MCV1 | Present study | ~7.0 × 10⁵ | ~4.7 × 10² cells/mm³ | ~1.1 × 10⁴ cells/mm³ | ~4.1 × 10⁵ ng/mL | ~5.6 × 10⁵ ng/mL | Comparable to the reference constructs; numerically higher simulated total-antibody peak than MCV2 |
|  | MCV2 | Present study | ~6.2 × 10⁵ | ~4.7 × 10² cells/mm³ | ~1.1 × 10⁴ cells/mm³ | ~4.1 × 10⁵ ng/mL | ~5.8 × 10⁵ ng/mL | Comparable to the reference constructs; numerically lower simulated total-antibody peak but slightly higher simulated IL-2 peak than MCV1 |
|  | MP_Pan2024_CTLA4_MEVC | Pan et al., 2024 | ~6.8 × 10⁵ | ~5.2 × 10² cells/mm³ | ~1.15 × 10⁴ cells/mm³ | ~4.2 × 10⁵ ng/mL | ~7.0 × 10⁵ ng/mL | Reference construct with strong simulated humoral and IL-2 responses |
|  | MP_Shahbazi2025_MEVC | Shahbazi et al., 2025 | ~6.5 × 10⁵ | ~5.2 × 10² cells/mm³ | ~1.1 × 10⁴ cells/mm³ | ~4.2 × 10⁵ ng/mL | ~5.8 × 10⁵ ng/mL | Reference construct with broadly comparable antibody, TH-cell, and cytokine patterns |
|  | CP_Noor2022_MEVC | Noor et al., 2022 | ~6.9 × 10⁵ | ~5.5 × 10² cells/mm³ | ~1.15–1.2 × 10⁴ cells/mm³ | ~4.2 × 10⁵ ng/mL | ~5.8 × 10⁵ ng/mL | Reference construct with high simulated antibody and TH-cell responses |
|  | CP_Guo2023_CTLA4_MEVC | Guo et al., 2023 | ~6.1 × 10⁵ | ~4.5 × 10² cells/mm³ | ~1.05–1.1 × 10⁴ cells/mm³ | ~4.2 × 10⁵ ng/mL | ~4.2 × 10⁵ ng/mL | Reference construct with slightly lower antibody/IL-2 peaks but comparable IFN-γ and TH-cell trends |
|  | **Table legend:** Values were extracted or visually estimated from deterministic C-ImmSim output plots generated using identical simulation settings. The symbol “~” indicates approximate values derived from graphical outputs. These values do not represent biological replicates, and no inferential statistical testing was performed. The comparison was conducted for descriptive in silico benchmarking only and should not be interpreted as experimental validation, evidence of immunological superiority, or prediction of actual vaccine efficacy. | | | | | | | |
